# Vitamin D receptor activation reduces VCaP xenograft tumor growth and counteracts ERG activity despite induction of TMPRSS2:ERG

**DOI:** 10.18632/oncotarget.17968

**Published:** 2017-05-18

**Authors:** Justin M. Roberts, Rebeca San Martin, D. Piyarathna Badrajee, James G. MacKrell, Guilherme V. Rocha, Jeffery A. Dodge, Cristian Coarfa, Venkatesh Krishnan, David R. Rowley, Nancy L. Weigel

**Affiliations:** ^1^ Department of Molecular and Cellular Biology, Baylor College of Medicine, Houston, TX, USA; ^2^ Alkek Center for Molecular Discovery, Baylor College of Medicine, Houston, TX, USA; ^3^ Lilly Research Labs, Eli Lilly and Company, Indianapolis, IN, USA; ^4^ Scott Department of Urology, Baylor College of Medicine, Houston, TX, USA

**Keywords:** vitamin D, prostate cancer, CYP24A1, TMPRSS2:ERG, VCaP

## Abstract

Whether vitamin D is chemopreventive and/or has potential therapeutically in prostate cancer is unresolved. One confounding factor is that many prostate cancers express a TMPRSS2:ERG fusion gene whose expression is increased both by androgens and by vitamin D receptor (VDR) activation. Two challenges that limit VDR agonist use clinically are hypercalcemia and the cooperation of VDR with ERG to hyper-induce the 1α,25-dihydroxyvitamin D_3_ metabolizing enzyme, CYP24A1, thus reducing VDR activity. Using the VCaP TMPRSS2:ERG positive cell line as a model, we found that a nonsecosteroidal CYP24A1 resistant VDR agonist, VDRM2, substantially reduces growth of xenograft tumors without inducing hypercalcemia. Utilizing next generation RNA sequencing, we found a very high overlap of 1,25D(OH)_2_D_3_ and VDRM2 regulated genes and by drawing upon previously published datasets to create an ERG signature, we found activation of VDR does not induce ERG activity above the already high basal levels present in VCaP cells. Moreover, we found VDR activation opposes 8 of the 10 most significant ERG regulated Hallmark gene set collection pathways from Gene Set Enrichment Analysis (GSEA). Thus, a CYP24A1 resistant VDR agonist may be beneficial for treatment of TMPRSS2:ERG positive prostate cancer; one negative consequence of TMPRSS2:ERG expression is inactivation of VDR signaling.

## INTRODUCTION

The role of vitamin D in chemoprevention or treatment of prostate cancer is controversial. Some epidemiological studies have found an inverse correlation between sunlight exposure or serum vitamin D metabolite levels and risk of prostate cancer or aggressive disease, but others have found no correlation or even an inverse correlation [1–8]. 1α,25-dihydroxyvitamin D_3_ (1,25D(OH)_2_D_3_) is the active vitamin D metabolite and is a ligand for the vitamin D receptor (VDR), a hormone activated transcription factor. VDR is widely expressed including in prostate and in prostate cancer. Treatment of prostate cancer cell lines with 1,25D(OH)_2_D_3_ typically is growth inhibitory *in vitro* although the extent of growth inhibition varies [9–11]. Inadequate dietary vitamin D results in elevated proliferation in mouse prostate epithelium [12] and some prostate cancer cell xenograft studies have shown a reduction in tumor growth upon VDR activation [13–15]. Despite promising pre-clinical results, clinical application of 1,25D(OH)_2_D_3_ has been disappointing with minimal to no effect reported [16, 17]. The best characterized physiological role for VDR is regulation of calcium and bone. Thus, one limitation of 1,25D(OH)_2_D_3_ treatment in cancer is the unacceptable side effect of hypercalcemia [18–20].

VDR action in prostate cancer has been studied in a limited number of models. About half of human prostate cancers contain a chromosomal rearrangement between the TMPRSS2 promoter and the coding region of an ETS transcription factor forming a TMPRSS2:ETS fusion gene [21]. The most common TMPRSS2:ETS fusion is TMPRSS2:ERG; this fusion promotes growth in prostate cancer cells, in mouse prostate, and in xenograft models [22–26]. TMPRSS2:ERG is induced by both the androgen receptor (AR) [21] and, as we have shown, VDR [10] raising the concern that VDR action in these tumors might be growth promoting rather than inhibitory. However, basal levels of ERG in fusion positive VCaP cells are 2000-fold higher than fusion negative LNCaP cells [21]. This raises the question of whether AR or VDR-mediated induction of TMPRSS2:ERG further increases ERG activity or if ERG activity already is maximal in these cells. As we have shown, one novel and potentially harmful effect of elevated ERG is its cooperation with VDR to hyper-induce the 1,25D(OH)_2_D_3_ metabolizing enzyme, CYP24A1, reducing levels of 1,25D(OH)_2_D_3_ and thus VDR activity [27]. We have shown that EB1089, a less calcemic 1,25D(OH)_2_D_3_ analog that is reported to be resistant to CYP24A1, inhibits growth of LNCaP xenograft tumors [13], but was unsuccessful in inhibiting growth of TMPRSS2:ERG expressing VCaP xenograft tumors [27]. This may have been due to an inability to deliver sufficient levels of agonist to reduce growth in the VCaP model *in vivo* without inducing hypercalcemia [27]. This left the question of whether any VDR agonist could inhibit growth of TMPRSS2:ERG positive cells *in vivo* unanswered.

In this study, we have tested a novel nonsecosteroidal VDR agonist, VDRM2, which has a large safety margin against hypercalcemia and is not predicted to be a substrate for CYP24A1 [28]. Although nonsecosteroidal agonists are less potent, VDRM2 was as efficacious in reducing growth of VCaP cells *in vitro* as was 1,25D(OH)_2_D_3_, it shared a nearly identical gene expression profile, and reduced VCaP tumor growth without inducing hypercalcemia *in vivo*. A comparison of our gene expression data with available data sets revealed that the level of ERG in VCaP cells was so high that neither treatment with 1,25D(OH)_2_D_3_ nor an androgen receptor agonist increased ERG target gene expression. Moreover, Gene Set Enrichment Analysis (GSEA) showed that treatment with 1,25D(OH)_2_D_3_ or VDRM2 counteracted the ERG dependent enrichment of c-Myc, E2F, and other Hallmark concepts.

## RESULTS

### VDRM2 induces VDR target genes and reduces growth of VCaP cells

**Figure 1:**
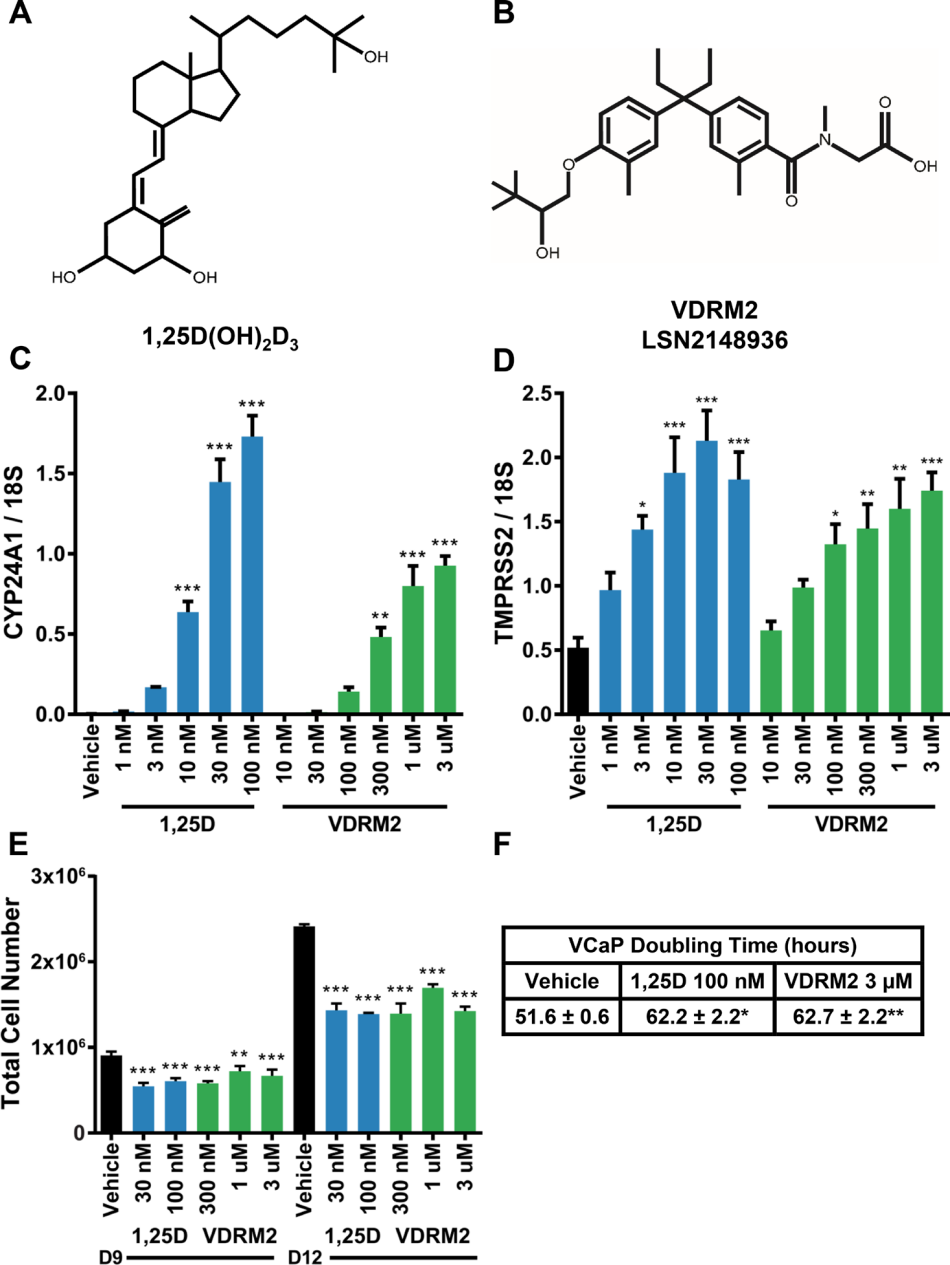
VDRM2 induces VDR target genes and reduces VCaP cell growth (**A**) Chemical structure of the active vitamin D metabolite 1α,25-dihydroxyvitamin D3 (1,25D(OH)_2_D_3_). (**B**) Chemical structure of non-secosteroidal VDR agonist, VDRM2 (LSN2148936). (**C**, **D**) VCaP cells were treated with the indicated doses of vehicle (EtOH), 1,25D(OH)_2_D_3_ (1,25D), or VDRM2 for 24 hours, harvested, and RNA was purified. VDR target genes CYP24A1 and TMPRSS2 were measured by RT-qPCR and normalized to 18S. (**E**) VCaP cells were treated with the indicated doses of vehicle (EtOH), 1,25D(OH)_2_D_3_, or VDRM2 for twelve days; medium and treatments were replenished every third day. Cells were counted using a Beckman-Coulter Counter. (**F**) Doubling time was calculated for vehicle (EtOH), 100 nM 1,25D(OH)_2_D_3_, or 3 μM VDRM2 treated cells from day 9 to day 12 from three independent experiments. *p < 0.05, **p < 0.01, ***p < 0.001, relative to vehicle control. *n* = 3, representative graph, mean ± SEM.

To determine the efficacy of VDRM2 in prostate cancer cells, we compared the activity of 1,25D(OH)_2_D_3_ and VDRM2. 1,25D(OH)_2_D_3_ is a secosteroid and is the active metabolite for VDR (Figure 1A). VDRM2 (LSN2148936) (Figure 1B) is a nonsecosteroidal VDR agonist that has been shown to restore bone mineral density in osteopenic, ovariectomized rats [28]. Although VDRM2 is much less potent than 1,25D(OH)_2_D_3_, the window between the concentration required to achieve a desired biological response in bone and the concentration that causes hypercalcemia is 57-fold for VDRM2 compared to 7.3-fold for 1,25D(OH)_2_D_3_ [28]. Treatment of VCaP cells with 1,25D(OH)_2_D_3_ or VDRM2 resulted in a dose dependent increase in mRNA of VDR target genes CYP24A1 (Figure 1C) and TMPRSS2 (Figure 1D). The EC_50_ values for CYP24A1 and TMPRSS2 were calculated based on Figure 1C and Figure 1D, respectively. For CYP24A1, the EC_50_ for 1,25D(OH)_2_D_3_ is 13.84 nM (Supplementary Figure 1A) and 298.54 nM for VDRM2 (Supplementary Figure 1B). For TMPRSS2, the EC_50_ for 1,25D(OH)_2_D_3_ is 1.89 nM (Supplementary Figure 1C) and 52.12 nM for VDRM2 (Supplementary Figure 1D). Fold change calculations for CYP24A1 induction by 1,25D(OH)_2_D_3_ are as follows, 10 nM (265×), 30 nM (604×), and 100 nM (722×) and for VDRM2, 300 nM (201×), 1 μM (333×), and 3 μM (386×) relative to vehicle control. Fold change calculations for TMPRSS2 for induction by 1,25D(OH)_2_D_3_ are as follows, 3 nM (2.8×), 10 nM (3.6×), 30 nM (4.1×), and 100 nM (3.5×) and for VDRM2, 100 nM (2.6×), 300 nM (2.8×), 1 μM (3.1×), and 3 μM (3.4×) relative to vehicle control. Additionally, using concentrations of agonist optimized for cell growth reduction, 1,25D(OH)_2_D_3_ and VDRM2 both reduce growth of VCaP cells at day 9 and day 12 post seeding (Figure 1E). Importantly, VDRM2 reduces growth of VCaP cells to the same extent as 1,25D(OH)_2_D_3_ although higher concentrations are needed as expected. Treatment with either VDR agonist increases VCaP cell doubling time from about 52 hours to about 62 hours (Figure 1F).

### VDRM2-dependent VDR activity is sustained in VCaP cells

**Figure 2:**
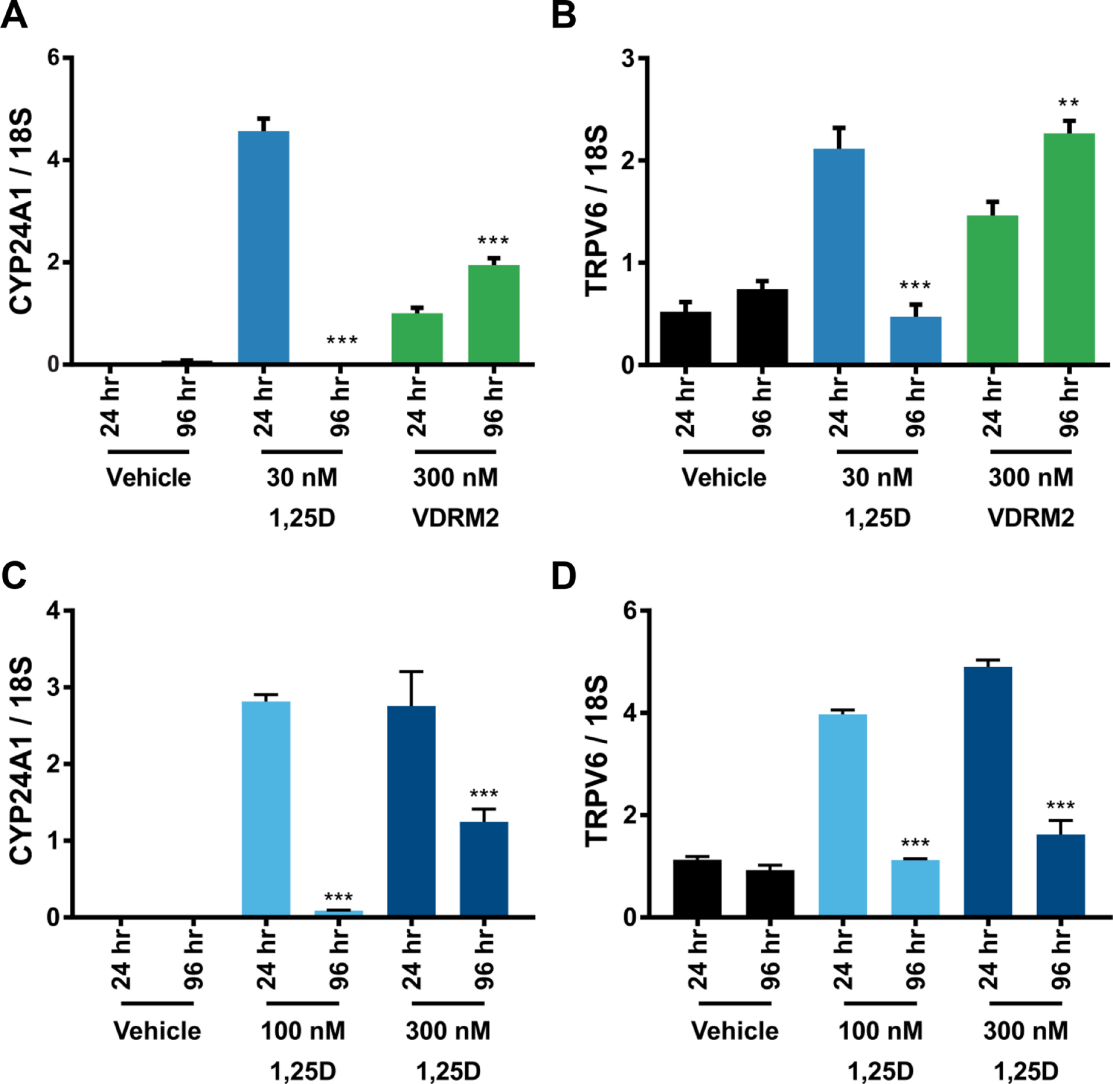
VDRM2-dependent VDR activity is sustained in VCaP cells (**A**, **B**) VCaP cells were treated with vehicle (EtOH), 30 nM 1,25D(OH)_2_D_3_ (1,25D) or 300 nM VDRM2 for 96 or 24 hours, harvested, and RNA was purified. VDR target genes CYP24A1 and TRPV6 were measured using RT-qPCR and normalized to 18S. (**C**, **D**) VCaP cells were treated with vehicle (EtOH), 100 nM or 300 nM 1,25D(OH)_2_D_3_ for 96 or 24 hours, harvested, and RNA purified. VDR target genes CYP24A1 and TRPV6 were measured using RT-qPCR and normalized to 18S. **p < 0.01, ***p < 0.001, relative to respective 24 hour time point. *n* = 3, representative graph, mean ± SEM.

**Figure 3:**
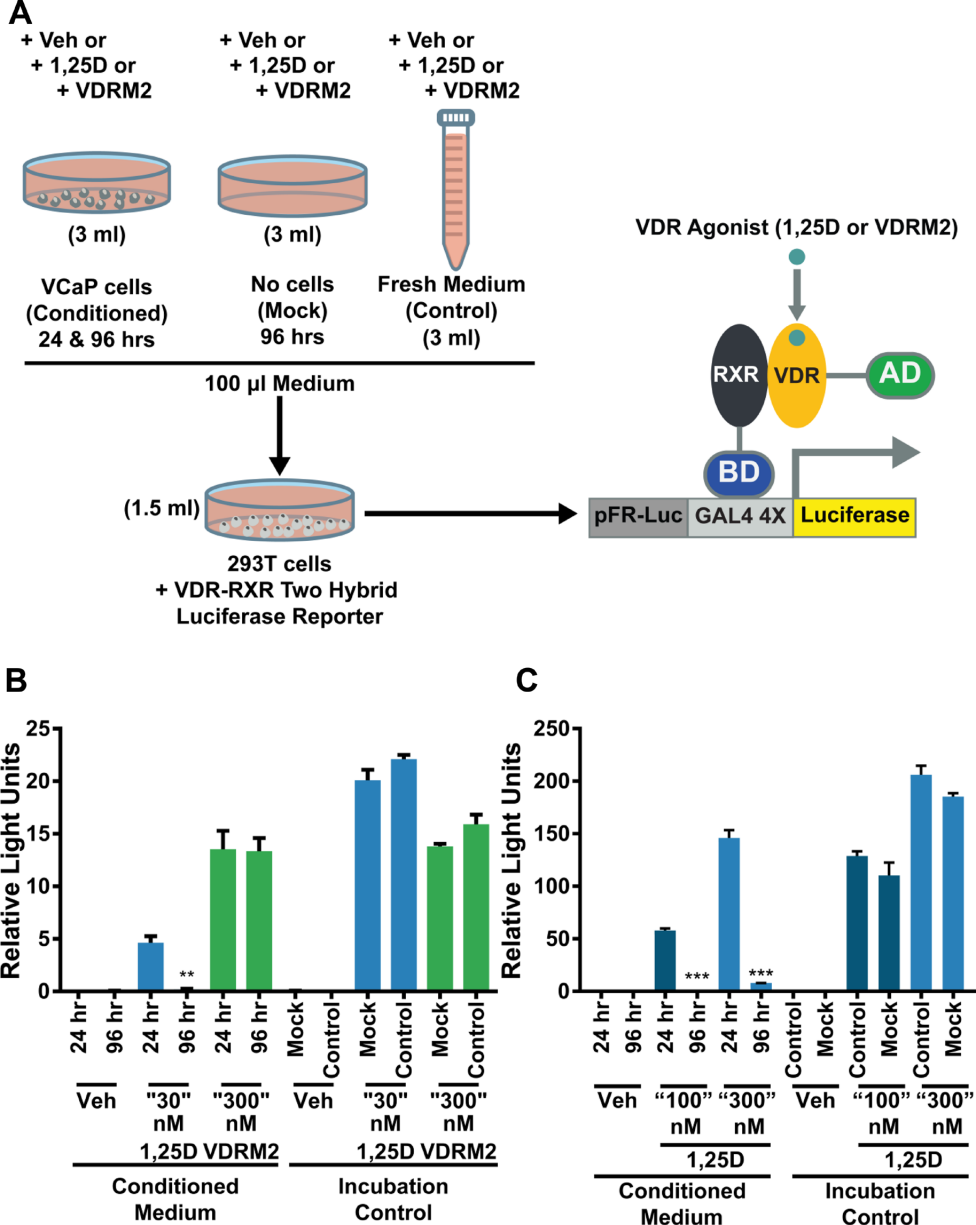
VDRM2 is not metabolized in VCaP cells (**A**) Diagram of the experimental procedure. VCaP cells were treated with vehicle (EtOH), 30 nM, 100 nM, or 300 nM 1,25D(OH)_2_D_3_ (1,25D), or 300 nM VDRM2 for 96 or 24 hours (Conditioned medium). Plates with no cells were incubated with the same concentrations of agonists for 96 hours (Mock) and fresh medium was prepared with the same concentrations of agonists at the end of the 96 hour incubation (Control). 293T cells were transfected with a VDR-RXR two-hybrid luciferase reporter as described in methods. A total of 100 μl of conditioned medium from VCaP cells in Figure 2 as well as media from the mock and control conditions were added to the transfected 293T cells in 1.5 ml of medium for 24 hours and then harvested. The cells were lysed and luciferase activity was measured and normalized to β-galactosidase activity. (**B**) Media assayed from VCaP cells treated with vehicle (EtOH), sub-maximum concentrations of 1,25D(OH)_2_D_3_ (30 nM), or 300 nM VDRM2 from Figure 2A, 2B. (**C**) Media assayed from VCaP cells treated with vehicle (EtOH) or higher concentrations of 1,25D(OH)_2_D_3_ (100 nM or 300 nM) from Figure 2C, 2D.**p < 0.01, ***p < 0.001, relative to respective 24 hour time point. *n* = 3, representative graph, mean ± SEM.

**Figure 4:**
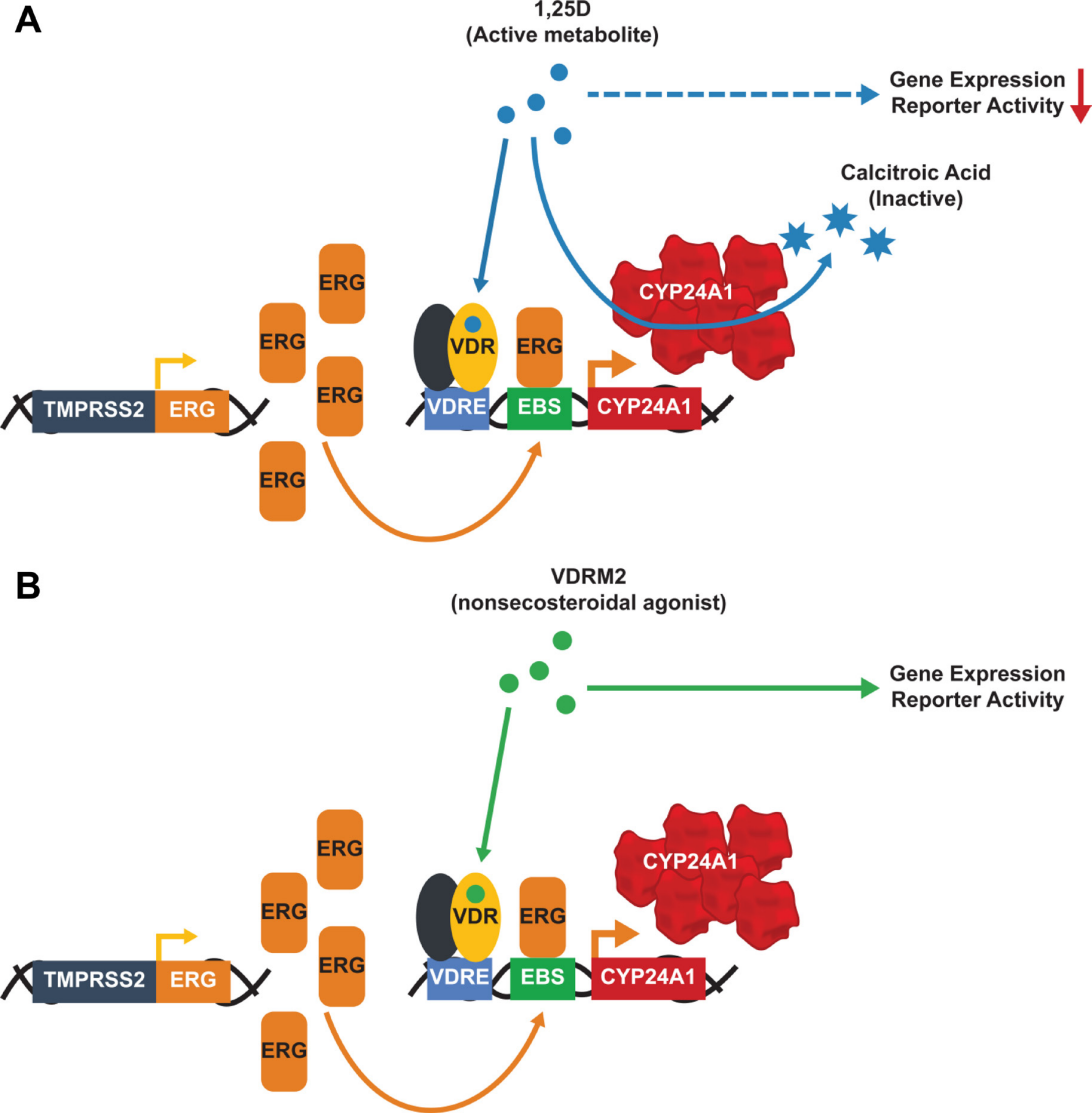
Hyper-induction of CYP24A1 limits the 1,25D(OH)_2_D_3_ but not the VDRM2-dependent actions of VDR The presence of the TMPRSS2:ERG fusion gene leads to high basal levels of ERG that cooperate with activated VDR to hyper-induce CYP24A1 expression in VCaP cells. (**A**) 1,25D(OH)_2_D_3_ (1,25D) is a substrate for CYP24A1 and 1,25D(OH)_2_D_3_-dependent VDR activity is reduced after incubation with VCaP cells. (**B**) VDRM2 is not predicted to be a substrate for CYP24A1 and VDRM2-dependent VDR activity is sustained after incubation with VCaP cells. VDRE: Vitamin D response element, EBS: Ets binding site.

We have shown previously that there is a hyper-induction of CYP24A1 mRNA levels in VCaP cells treated with 1,25D(OH)_2_D_3_ due to collaboration of ERG with VDR; consequently, 1,25D(OH)_2_D_3_-dependent VDR activity is reduced in a time-dependent manner consistent with metabolism of 1,25D(OH)_2_D_3_ [27]. VDRM2 is structurally dissimilar to 1,25D(OH)_2_D_3_ and would not be predicted to be a substrate for CYP24A1. To determine whether VDRM2 is metabolized or inactivated in VCaP cells, the levels of VDR-mediated gene expression were measured as a surrogate for stability of VDR agonists. VCaP cells were treated with sub-maximum doses of either 1,25D(OH)_2_D_3_ or VDRM2 for 96 and 24 hours and RNA was isolated. In contrast to 1,25D(OH)_2_D_3_, which lost activity at 96 hours, VDRM2-dependent VDR activity is increased in a time dependent manner as measured by increased mRNA expression of VDR target genes CYP24A1 (Figure 2A) and TRPV6 (Figure 2B). To determine whether the difference observed between 1,25D(OH)_2_D_3_ and VDRM2 was due to differences in agonist concentration, the assay was also completed using 100 nM and 300 nM 1,25D(OH)_2_D_3_ and again 1,25D(OH)_2_D_3_-dependent VDR activity was reduced in a time-dependent manner measured by decreased mRNA expression of VDR target genes CYP24A1 (Figure 2C) and TRPV6 (Figure 2D). To further examine remaining activity of the VDR agonists after incubation with VCaP cells, we assayed the residual VDR agonist in the medium. 293T cells were transfected with plasmids for an agonist dependent VDR-RXR two-hybrid luciferase reporter and treated with conditioned medium from the VCaP cells or hormones incubated with medium alone (see Figure 3A for a schematic of the assay and methods). We found that VDRM2-dependent VDR activity is maintained after incubation with VCaP cells whereas 1,25D(OH)_2_D_3_-dependent activity is significantly reduced at sub-maximum (30 nM) (Figure 3B) and higher concentrations (100 nM or 300 nM) of 1,25D(OH)_2_D_3_ (Figure 3C). Neither 1,25D(OH)_2_D_3_ nor VDRM2-dependent VDR activity is lost when the ligands are incubated without cells (mock) suggesting the ligands are stable within the times tested and the maintenance of VDRM2-dependent signaling is due to lack of metabolism. As depicted in our model, these findings suggest that high basal levels of ERG, due to the TMPRSS2:ERG fusion gene, cooperate with VDR on the CYP24A1 promoter to hyper-induce CYP24A1 expression leading to reduced 1,25D(OH)_2_D_3_-dependent VDR activity and signaling (dashed arrow) (Figure 4A). In contrast, VDRM2 is not inactivated by the increased expression of CYP24A1 and therefore VDRM2-dependent VDR activity and signaling remains intact (Figure 4B).

### VDRM2 reduces growth of VCaP xenograft tumors

**Figure 5:**
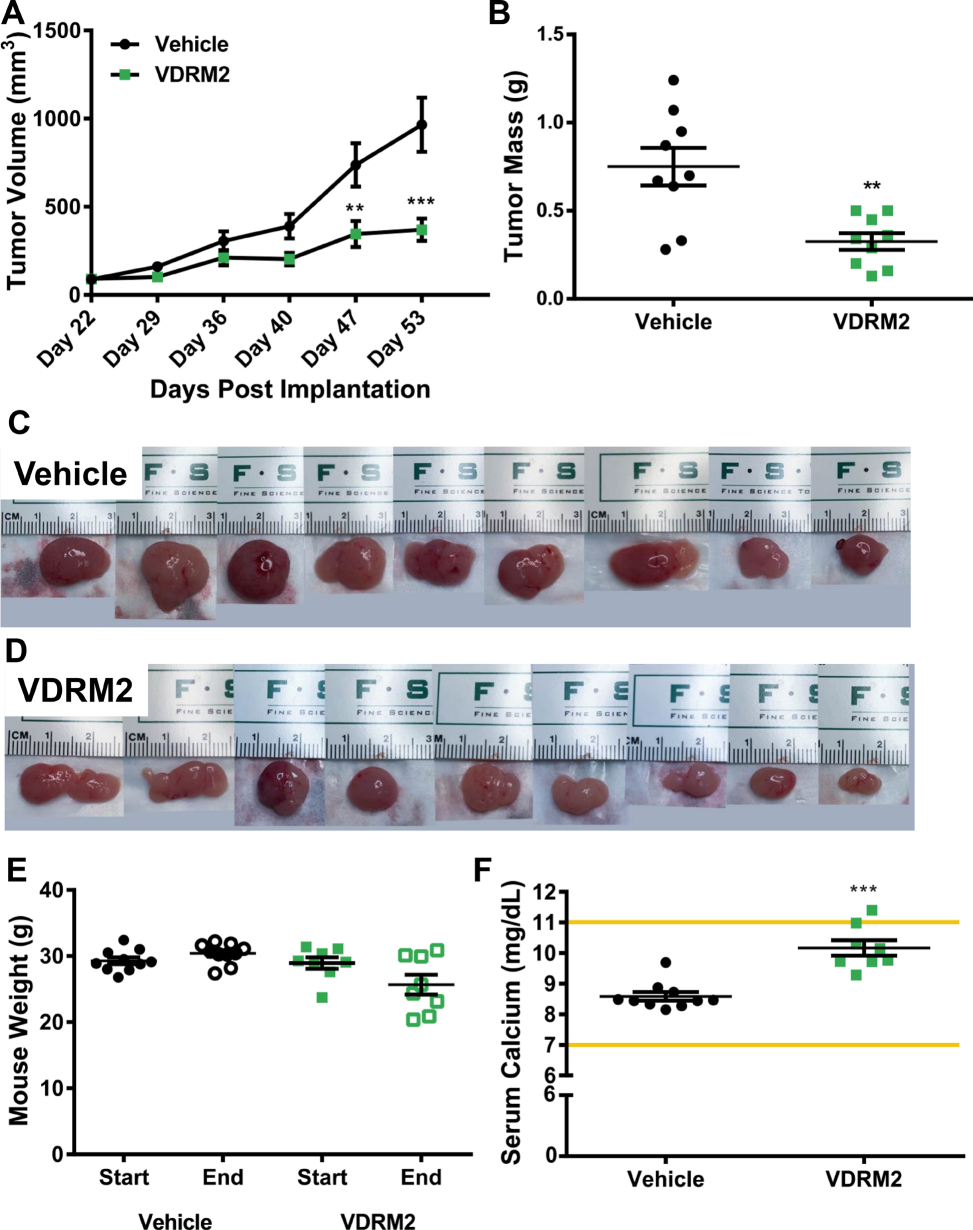
VDRM2 reduces tumor growth in a VCaP xenograft model (**A**) Tumor measurements were taken using digital calipers once per week starting on Day 22 when the tumors were palpable for measuring. *n = 9* vehicle, *n = 9* VDRM2. (**B**) At the end of the study mice were euthanized and tumors were removed and weighed using a digital balance. *n = 9* vehicle, *n = 9* VDRM2. (**C**) Images of the tumors harvested from vehicle-treated mice. (**D**) Images of the tumors harvested from VDRM2-treated mice. (**E**) Mice were weighed at the start and end of the study and weights plotted. *n = 10* vehicle, *n = 8* VDRM2. (**F**) Under deep anesthesia mice were exsanguinated via cardiac puncture and serum was separated from the blood. Serum samples were sent to the Baylor College of Medicine Pathology Core for serum calcium analysis. *n = 10* vehicle, *n = 8* VDRM2. .**p < 0.01, ***p < 0.001, mean ± SEM.

Whether a VDR agonist can reduce the growth of a prostate cancer cell line containing a TMPRSS2:ERG rearrangement *in vivo* has not been resolved. To address this deficiency, we used the VCaP cell line in the DRS xenograft model (a combination of prostate cancer cells, prostate stromal cells (HPS-19I), and Matrigel) [29]. Twenty male NCr Nude mice were randomly assigned to either vehicle (sesame oil), or VDRM2 (3 μg/kg) groups. VCaP cells and HPS-19I cells were mixed with Matrigel and injected subcutaneously into the left and right flank of each mouse. The day after injection of the tumors, mice started treatment via oral gavage five days per week. VDRM2 treated mice had smaller average tumor volumes at day 47 and 53 post implantation (Figure 5A) and significantly reduced tumor mass (Figure 5B). Images of tumors from vehicle (Figure 5C) and VDRM2-treated (Figure 5D) mice display marked size differences. Compared to the initial measurement, there was no statistically significant reduction in average start versus end weights of the treated mice (Figure 5E). A critical limitation of studies using VDR agonists is the possibility of hypercalcemia. Serum calcium levels were significantly elevated in VDRM2-treated mice; however, the average serum calcium level for VDRM2 treated mice was within the normal range indicated by the lines on the graph (Figure 5F). To determine whether VDRM2 is acting systemically, we measured renal Cyp24a1 mRNA and found significant induction in VDRM2-treated mice as expected (Supplementary Figure 2).

### RNA-sequencing reveals extensive overlap of 1,25D(OH)_2_D_3_ and VDRM2 regulated genes and GSEA shows that c-Myc signaling is a major target of VDR action

**Figure 6:**
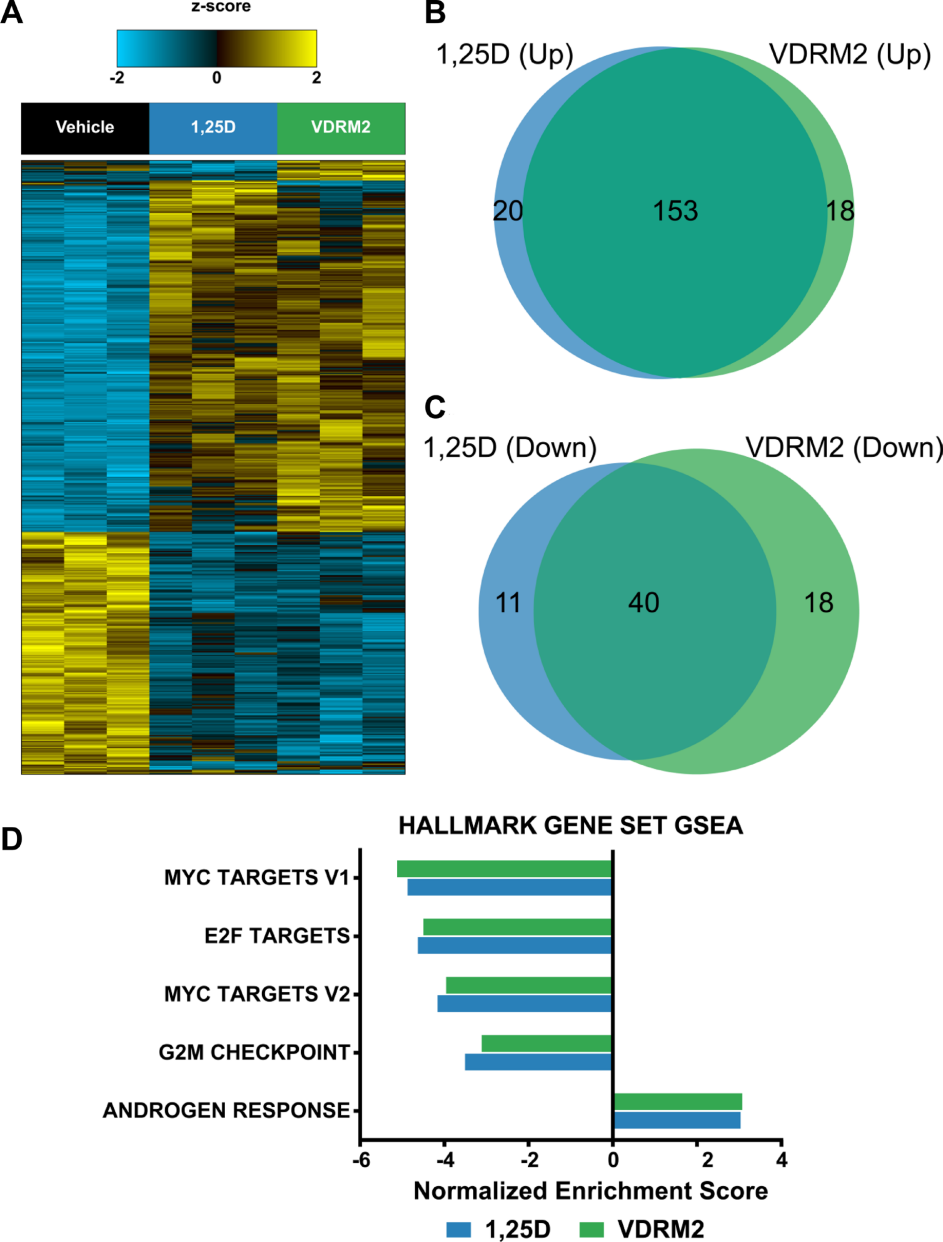
RNA sequencing reveals extensive overlap of 1,25D(OH)_2_D_3_ and VDRM2-regulated genes and GSEA recapitulates known VDR target pathways in prostate cancer VCaP cells were treated with vehicle,10 nM 1,25D(OH)_2_D_3_ (1,25D), or 1 μM VDRM2 for 24 hours and RNA was purified. Transcriptomic profiling was assessed using RNA-Seq on an Illumina HiSeq2000. (**A**) Heat map showing an ANOVA comparison of vehicle, 1,25D(OH)_2_D_3_, or VDRM2-treated VCaP cells (q < 0.25). (**B**) Venn diagram comparing genes upregulated at least 1.5-fold by 1,25D(OH)_2_D_3_ or VDRM2. (**C**) Venn diagram comparing genes downregulated at least 1.5-fold by 1,25D(OH)_2_D_3_ or VDRM2. (**D**) The top 5 pathways significantly regulated via GSEA using the Hallmark gene set collection, sorted in ascending order by the normalized enrichment score (NES).

We used next generation sequencing to compare the transcriptome of 1,25D(OH)_2_D_3_ and VDRM2-treated VCaP cells at concentrations of ligands that yielded comparable levels of CYP24A1 induction in pilot analyses. A heat map shows 1,25D(OH)_2_D_3_ and VDRM2 mediated gene expression is very similar (Figure 6A). Comparing the numbers of genes upregulated (Figure 6B) and downregulated (Figure 6C) at least 1.5-fold reveals a greater than 80% overlap of induced VDR target genes. An examination of the “unique” genes revealed that most were also regulated, but fell below the 1.5-fold cut off increasing the percent regulated by both ligands to > 90% (Supplementary Tables 1, 2, 3, 4). Lists of the top 30 genes upregulated or down regulated by 1,25D(OH)_2_D_3_ (Supplementary Tables 5, 6) and VDRM2 (Supplementary Tables 7, 8) have been provided. The list contains a number of previously identified VDR regulated genes including CYP24A1, SULT1C2, TRPV6, TMPRSS2, and CEBPδ as well as a number of novel genes. To query pathways regulated by VDR ligand treatment, Gene Set Enrichment Analysis (GSEA) was completed. Analysis of the top 5 Hallmark gene set pathways shows a reduction in c-Myc and E2F pathways by both agonists consistent with our understanding of VDR signaling in the LNCaP lineage of prostate cancer cells [30, 31]. This further confirms the similarity of pathways regulated by 1,25D(OH)_2_D_3_ and VDRM2, and suggests our dataset is a useful tool for further inquiry into VDR regulated pathways (Figure 6D) (see Supplementary Table 9 for normalized enrichment scores and *q*-values).

### Treatment of VCaP cells with VDR agonist does not induce an ERG target gene signature

**Figure 7:**
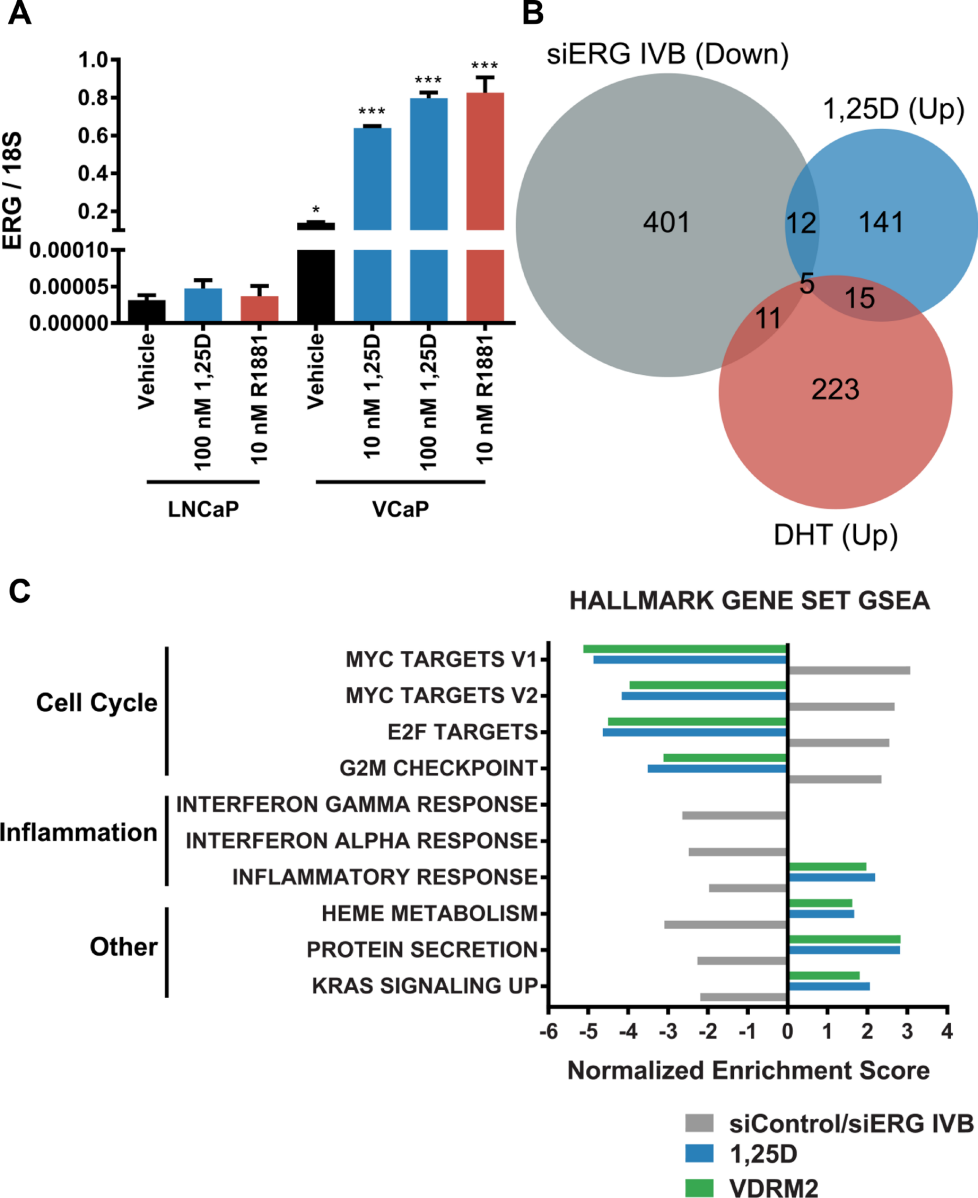
Defined ERG target gene signature is not increased in 1,25D(OH)_2_D_3_-treated VCaP cells (**A**) LNCaP or VCaP cells were changed to stripped serum and treated for 24 hours with vehicle (EtOH), 10 nM 1,25D(OH)_2_D_3_ (1,25D), 100 nM 1,25D(OH)_2_D_3_, or 10 nM R1881 and RNA was purified. ERG mRNA was measured by RT-qPCR and normalized to 18S. (**B**) Venn diagram showing comparison of a previously published microarray from VCaP cells treated with siRNA against ERG (siERG IVB), a previously published microarray from VCaP cells treated with DHT, and our RNA-seq data from VCaP cells treated with 10 nM 1,25D(OH)_2_D_3_. The siERG IVB group denotes genes significantly downregulated at least 1.5-fold relative to control siRNA (genes requiring ERG for expression) and the DHT and 1,25D(OH)_2_D_3_ groups denote genes significantly upregulated at least 1.5-fold relative to vehicle control. (**C**) The top 10 pathways significantly regulated via GSEA using the Hallmark gene set collection comparing siERG IVB (ERG induced),1,25D(OH)_2_D_3_, and VDRM2-treated VCaP cells plotted as normalized enrichment score. *p < 0.05, ***p < 0.001, relative to respective LNCaP treatment. *n* = 3, representative graph, mean ± SEM.

We have previously reported that despite induction of ERG, 1,25D(OH)_2_D_3_ reduces growth of VCaP cells [10]. However, we have not addressed whether 1,25D(OH)_2_D_3_ induces a pattern of gene expression consistent with an ERG target gene signature. Comparatively, VCaP cells express several orders of magnitude higher ERG mRNA than LNCaP cells at basal levels and there is no induction of ERG in LNCaP cells with 1,25D(OH)_2_D_3_ or R1881 (Figure 7A). Consistent with this, in previous studies (Kim et al. ref Figure 4B), we found that although ERG protein was highly expressed in VCaP and somewhat further induced by 1,25D(OH)_2_D_3_, ERG protein was undetectable in LNCaP cells [27]. By drawing upon previously published datasets as well as our own, we have created an ERG signature. The ERG dataset (E-MTAB-2838) is from VCaP cells treated with siRNA targeting the type IV TMPRSS2:ERG fusion variant for 48 hours [32], the DHT dataset (GSE62473) is from VCaP cells treated with dihydrotestosterone (DHT) for 24 hours, and the 1,25D(OH)_2_D_3_ dataset is from our RNA-seq from VCaP cells treated with 1,25D(OH)_2_D_3_ for 24 hours. A Venn diagram shows the number of genes significantly downregulated upon treatment with siRNA targeting ERG (siERG IVB) (i.e. ERG induced), the number of genes significantly upregulated by 1,25D(OH)_2_D_3_ treatment, and the number of genes significantly upregulated by DHT treatment in VCaP cells (Figure 7B). We formed the ERG signature based on the genes that were significantly downregulated in the siERG IVB group versus control siRNA treated cells. By comparing this signature to the genes significantly upregulated by 1,25D(OH)_2_D_3_ or DHT versus vehicle treatment, we found that of the ERG-regulated genes, only 3.9% overlapped with 1,25D(OH)_2_D_3_-regulated genes and 3.7% overlapped with DHT-regulated genes. This suggests that the basal amount of ERG in VCaP cells is high enough to induce the ERG target gene signature and the additional increase in ERG transcript levels with either 1,25D(OH)_2_D_3_ or DHT does not have a significant impact on expression of ERG target genes. Furthermore, by comparing GSEA from siRNA control vs ERG IVB to 1,25D(OH)_2_D_3_ or VDRM2-treated VCaP cells, we found treatment with either ligand inversely correlates with 8 of the 10 top ERG-regulated Hallmark gene set concepts suggesting that despite the induction of ERG transcript levels, 1,25D(OH)_2_D_3_ or VDRM2 treatment has a net negative effect on pathways regulated by ERG (Figure 7C) (see Supplementary Table 10 for normalized enrichment scores and *q*-values).

## DISCUSSION

Despite some promising *in vitro* and pre-clinical studies, the capacity of activated VDR to inhibit prostate cancer growth in humans is unclear. There are at least two major challenges in using VDR activation as a therapy in prostate cancer. The first is inducing sufficient activation of tumor VDR without adverse systemic side effects. VDR regulates calcium metabolism [33] and high levels of 1,25D(OH)_2_D_3_ result in hypercalcemia [34]. Efforts to identify VDR ligands that are relatively more potent than 1,25D(OH)_2_D_3_ in inducing activities in specific tissues or in tumors relative to their ability to induce hypercalcemia have yielded some candidates although all retain the capacity to induce hypercalcemia [35–37]. Despite this caveat, these may be more useful than 1,25D(OH)_2_D_3_ (calcitriol). A second confounding factor in comparing the pre-clinical studies with studies in humans is that more than 50% of human tumors contain the TMPRSS2:ERG rearrangement. We have shown that 1,25D(OH)_2_D_3_ increases expression of TMPRSS2:ERG [10] and that ERG acts synergistically with VDR to hyper-induce CYP24A1, the enzyme that inactivates 1,25D(OH)_2_D_3_ [27]. Depletion of ERG in VCaP cells reduced VDR mediated induction of CYP24A1 in VCaP cells without altering induction of other VDR target genes [27]. Moreover, lentiviral mediated expression of ERG in LNCaP cells also greatly increased the VDR-mediated induction of CYP24A1 showing that this is not limited to one cell background [27]. TMPRSS2:ERG is growth promoting in VCaP prostate cancer cells and in mouse models [23–25], although whether androgen or 1,25D(OH)_2_D_3_ induction of the already highly expressed TMPRSS2:ERG increases ERG activity had not been examined.

There is evidence that a high level of CYP24A1 and/or other factors that limit tumor levels of 1,25D(OH)_2_D_3_ can prevent VDR mediated growth inhibition [38, 39]. CYP24A1 is overexpressed in prostate cancer [39]. In the DU145 prostate cancer cell line, CYP24A1 is overexpressed causing resistance to 1,25D(OH)_2_D_3_-mediated growth inhibition unless CYP24A1 activity is blocked by inhibitors [38, 40]. Furthermore, a reduction of CYP24A1 expression in DU145 xenograft tumors results in robust 1,25D(OH)_2_D_3_-mediated growth inhibition *in vivo* in an otherwise recalcitrant cell line [39]. Systemic inhibition of CYP24A1 can result in hypercalcemia and thus we sought to identify a CYP24A1 resistant VDR agonist, which could be given in sufficient quantities to reduce VCaP xenograft growth. In our previous study, the less calcemic, CYP24A1 resistant 1,25D(OH)_2_D_3_ analog, EB1089, did not reduce VCaP xenograft tumor growth *in vivo* despite cell growth reduction *in vitro* [27] and efficacy in LNCaP xenograft tumors [13]. This discrepancy in action between *in vitro* VCaP cell growth and *in vivo* xenograft tumor efficacy of EB1089 in the VCaP model is likely due to the limitation of the amount of EB1089 given due to risk of hypercalcemia *in vivo*. *In vitro*, LNCaP cells are more responsive to VDR-mediated growth inhibition than are VCaP cells [27] and also responded to EB1089 *in vivo* [13].

An alternative approach is to utilize nonsecosteroidal VDR agonists that are not predicted to be substrates for CYP24A1 metabolism and show greater discrimination between levels required to induce a desired biological response and those that induce hypercalcemia. A nonsecosteroidal VDR agonist, LG190119, has been shown previously to inhibit growth of LNCaP xenograft tumors *in vivo* without causing hypercalcemia [15]. We have tested a newer nonsecosteroidal VDR agonist, VDRM2, from Lilly that has been shown to restore bone mineral density in osteopenic, ovariectomized rats [28]. Although it is a less potent agonist than 1,25D(OH)_2_D_3_ [28], in VCaP cells it induces VDR target genes (Figure 1C, 1D) and, most importantly, reduces cell growth equivalently (Figure 1E) although at higher concentrations than 1,25D(OH)_2_D_3_. In order to determine the stability of VDRM2 in cells that overexpress CYP24A1, we used two different biological assays and found by VDR target gene induction (Figure 2A, 2B) or activation of a VDR agonist-dependent VDR-RXR two-hybrid reporter (Figure 3B), that VDRM2 is stable and is not lost due to other, unanticipated mechanisms. In contrast, 1,25D(OH)_2_D_3_ was clearly inactivated presumably through sequential modifications by CYP24A1 (Figure 2A–2D; Figure 3B, 3C).

VDRM2 substantially reduced VCaP xenograft growth without significant adverse effects. Moreover, in this experiment, the mice were maintained on a diet containing 200 IU (5 μg/kg) of dietary vitamin D shown to induce human relevant serum 25-hydroxvitamin D_3_ levels in mice [12]. Therefore, the effect on tumor growth, while significant, might have been even greater if we mimicked human vitamin D deficiency. Nevertheless, our study is the first to show that a VDR agonist reduces growth of TMPRSS2:ERG positive xenograft tumors *in vivo* (Figure 5A–5D). Importantly, VDRM2 did not induce hypercalcemia in most of the treated mice (Figure 5F). Although some mice lost weight, the average weight loss was not statistically significant (Figure 5E). Whether a lower dose of VDRM2 would be equally efficacious in reducing tumor growth with less elevation of calcium is worth testing. Previous studies have shown nonsecosteroidal VDR mimics do not bind well to human or rodent serum vitamin D binding protein (DBP) and it is this attribute that has been suggested to play a role in decreased calcium mobilization [41, 42]. It is important to note we used an athymic mouse model to complete our xenograft studies and thus VDR actions on some components of the immune system were not present in this model. There is great interest in and some success of immune-related therapies [43–46] in cancer. VDR regulates T cells [47], dendritic cells [48, 49], and the reprogramming of pancreatic stromal cells to reduce markers of inflammation [50]. Some of these actions may improve response of tumors to VDR agonists, while others may limit response. This could be addressed, in future by using more advanced humanized mouse models [51–53] to study the immune modulatory effects of VDR activation.

Transcriptome analysis of VDR signaling in prostate cancer cells has been limited to microarray analysis [54–58] and has not included a TMPRSS2:ERG positive cell line. Our study is the first to explore VDR action in prostate cancer using next generation RNA sequencing of the TMPRSS2:ERG positive VCaP cell line. The overlap between 1,25D(OH)_2_D_3_ and VDRM2 regulated genes (Figure 6B, 6C) including those that are regulated in the same direction, but fall below the 1.5-fold cut-off is extremely high. This suggests that the mechanism for the enhanced window between inducing VDR activity and causing hypercalcemia is not due to selective activation of a sub-set of VDR target genes within the prostate cancer cell. Rather, it likely is due to systemic differences in cell specific uptake, half-life in circulation, and/or actions in other tissues. There is little agreement as to which genes are induced by 1,25D(OH)_2_D_3_ in prostate cancer cells with discrepancies between cell lines and even between studies in the same cell type. In this study, we detect induction of CYP24A1 and TRPV6, two VDR target genes regulated in most tissues. Other genes, which have previously been reported to be regulated in LNCaP cells including CEBPδ [59], TMPRSS2 [10], NDRG1 [58], and c-Myc [31] are also found in our data set. However, under the conditions used, we did not detect IGFBP3 regulation [60], for example. Analysis of the Hallmark gene set concepts shows that down-regulation of Myc and E2F targets are the three most significant concepts from GSEA. This is consistent with our finding in the LNCaP/C4-2 lineage that the down-regulation/inactivation of c-Myc mimics the 1,25D(OH)_2_D_3_-mediated growth inhibition [31]. Because c-Myc induces E2F family members, it is not surprising that E2F concepts are reduced. The fourth category is a reduction in G2/M. In some cell lines, 1,25D(OH)_2_D_3_ causes a G1 arrest and a reduction in c-Myc also will cause G1 accumulation. The fifth most significant concept is induction of an androgen response. In normal prostate, the role of androgen in the epithelial cells is differentiating and to produce prostate specific secreted proteins such as PSA. Figure 7B shows that about 11% of the VDR-regulated genes also are regulated by androgen receptor.

The question of whether VDR agonist would be beneficial or harmful in TMPRSS2:ERG positive prostate cancer has been an important question since we observed that VDR induces its expression [10]. Even in hormone depleted media, basal levels of ERG in VCaP cells are orders of magnitude higher than in TMPRSS2:ERG negative LNCaP cells (Figure 7A). The addition of either R1881 or 1,25D(OH)_2_D_3_ increases ERG expression only about 4-fold over basal levels in VCaP cells (Figure 7A). To test whether 1,25D(OH)_2_D_3_-mediated induction of TMPRSS2:ERG resulted in more ERG activity, we compared the 1,25D(OH)_2_D_3_ induced genes with a set of genes whose expression is reduced when ERG is reduced (ERG regulated genes). Because androgen receptor also induces TMPRSS2:ERG, we similarly compared with androgen (DHT) regulated genes. As shown in Figure 7B, there was very little overlap of the 1,25D(OH)_2_D_3_ or DHT signature with the ERG signature. From these observations we conclude there is no significant induction of ERG activity upon the addition of hormone in VCaP cells and the majority of ERG activity is likely from high basal levels of ERG expression possibly due to the open chromatin nature of the region around the TMPRSS2:ERG fusion [61]. One possible limitation of our study is the heterogeneity of TMPRSS2:ERG expression in prostate cancer patient tumors [62–65]. This is particularly important given that the VCaP cell line expresses high basal levels of ERG and addition of 1,25D(OH)_2_D_3_ or R1881 modestly increases ERG RNA levels but does not induce ERG activity. Given that the patient data demonstrate heterogeneity of ERG expression in TMPRSS2:ERG positive prostate cancer, we cannot completely exclude the possibility that VDR-mediated induction of ERG will cause an increase in ERG activity in cells with low basal levels of TMPRSS2:ERG expression. However, activation of VDR also counteracts many of the actions of ERG. Figure 7C shows the 10 most significant Hallmark GSEA ERG concepts. Remarkably, 1,25D(OH)_2_D_3_ and VDRM2 have the opposite effect on 8 out of 10 categories and no effect in the other two inflammation-based concepts. Included in the most significant for ERG are the same Myc and E2F concepts that were found to be most significant for 1,25D(OH)_2_D_3_ and VDRM2.

In summary, we have identified VDRM2 as a VDR agonist that overcomes two challenges to VDR agonist use clinically. VDRM2 significantly reduces TMPRSS2:ERG positive VCaP xenograft tumor growth without causing hypercalcemia or significant weight loss in mice and VDRM2 is stable in cells that overexpress CYP24A1. Moreover, at least in this model, the VDR-mediated induction of TMPRSS2:ERG does not increase ERG activity. Remarkably, VDR counteracts many of the actions of ERG. We speculate that one of the harmful actions of ERG is the synergistic induction of CYP24A1 reducing endogenous VDR activity and that a CYP24A1 resistant VDR agonist may be useful in patients with TMPRSS2:ERG positive tumors.

## MATERIALS AND METHODS

### Materials and reagents

The VDR agonist 1α,25-dihydroxyvitamin D3, (1,25D(OH)_2_D_3_) was purchased from Selleck Chemicals (Houston, TX). LSN2148936 (VDRM2) was graciously provided by Eli Lilly and Company (Indianapolis, Indiana) [28]. Agonists were dissolved in 100% ethanol and stored at −20°C protected from light. Vehicle treatments contained no more than 0.1% ethanol. Unless specifically described, all other reagents were a minimum of reagent grade.

### Cell lines

VCaP cells were purchased from the American Type Culture Collection (Manassas, VA) and cultured in DMEM F12 1:1 (ThermoFisher, Waltham, MA) containing 10% fetal bovine serum from Sigma-Aldrich (St. Louis, MO). LNCaP cells were purchased from the American Type Culture Collection and cultured in RPMI 1640 (ThermoFisher, Waltham, MA) containing 10% fetal bovine serum. 293T cells were purchased from the American Type Culture Collection and cultured in DMEM (ThermoFisher, Waltham, MA) containing 10% fetal bovine serum. HPS-19I cells were cultured in BFS medium (90% DMEM, 5% fetal calf serum, 5% Nu-Serum, penicillin (100 units/ml), streptomycin (100 μg/ml), insulin (5 μg/ml), and testosterone (0.5 μg/ml) [66]. Cells were cultured in a humidified incubator at 5% CO_2_ and were cultured for not more than three months before restarting passage. All cells have undergone short tandem repeat (STR) analysis and are routinely tested for mycoplasma. All cell experiments contained three independent samples/treatment and each experiment was completed a minimum of three times unless otherwise indicated.

### Growth assay

VCaP cells were plated at 100,000 cells per well in 6-well plates (Corning, Tewksbury, MA). Cells were treated with vehicle (EtOH), 30 nM 1,25D(OH)_2_D_3_, 100 nM 1,25D(OH)_2_D_3_, 300 nM VDRM2, 1 μM VDRM2 or 3 μM VDRM2. Media and treatments were changed every three days. Cell counts were measured using a Beckman-Coulter Counter on Day 0, Day 9, and Day 12 after plating. Doubling time was calculated for vehicle (EtOH), 100 nM 1,25D(OH)_2_D_3_, or 3 μM VDRM2 treated cells from day 9 to day 12. The formula: *time x log(2)/ log(Final) – log(Initial)* was used to calculate doubling time.

### Stability of VDR agonists

Treatment of cells with VDR agonists: VCaP cells were plated at 400,000 cells per well in 6-well plates. Cells were allowed to adhere for 4 days. Media was replenished and some of the cells were treated with vehicle (EtOH), 30 nM, 100 nM, or 300 nM 1,25D(OH)_2_D_3_, or 300 nM VDRM2 for 96 hours. Others were treated 72 hours post medium replenishment with vehicle (EtOH), 30 nM, 100 nM, or 300 nM 1,25D(OH)_2_D_3_, or 300 nM VDRM2 for 24 hours. For the incubation controls, the mock condition was prepared by adding 3 ml of medium to 6-well plates containing no cells and treating with vehicle (EtOH), 30 nM, 100 nM, or 300 nM 1,25D(OH)_2_D_3_, or 300 nM VDRM2 for 96 hours and the control condition was prepared from 3 ml of media containing vehicle (EtOH), 30 nM, 100 nM, or 300 nM 1,25D(OH)_2_D_3_, or 300 nM VDRM2. All cells and conditioned media were harvested at the end of 96 hours. RNA was isolated from the cells and VDR target genes measured. The medium from all conditions was collected and the remaining agonist in the medium was assayed using a mammalian two-hybrid assay, which measures agonist dependent interaction of VDR and its heterodimer partner, RXR. Briefly, 293T cells were transfected with 10 ng pCMV-AD-hVDR (prey), 30 ng pCMV-BD-hRXRα (bait), 500 ng pFR-Luc reporter gene (gifts of Dr. Peter Jurutka) [67] and 100 ng pCR 3.1 β-galactosidase expression plasmid [68] using Lipofectamine 2000 (ThermoFisher, Waltham, MA). After 24 hours, the medium was replenished. A total of 100 μl of medium from the treated VCaP cells was added to a corresponding well of the 293T cells containing 1.5 ml of fresh medium. Additionally, 100 μl of medium from the mock condition as well as 100 μl from the control condition was added to a corresponding well of 293T cells. After 24 hours, the 293T cells were harvested in ice-cold phosphate buffered saline (PBS) (ThermoFisher, Waltham, MA), collected by centrifugation for 1 minute at 13,000 rpm, and the PBS was aspirated. Cells were lysed in 200 μl of Reporter Lysis Buffer (Promega, Madison, WI) containing protease inhibitors (GenDepot, Barker, TX) by vortexing the cells until resuspended and completing one freeze/thaw cycle at −80°C. Lysates were centrifuged at 13,000 rpm for 1 minute. Luciferase activity was measured from 100 μl of lysate using the Luciferase Assay System (Promega, Madison, WI) and β-galactosidase activity was measured using 50 μl of lysate as previously described [69]. The data are reported as luciferase/β-galactosidase activity and expressed as relative light units.

### Quantitative RT-PCR

RNA was prepared using TriReagent (GenDepot, Barker, TX). A total of 0.5 μg of RNA was converted to cDNA using amfiRivert cDNA Synthesis Platinum Master Mix (GenDepot, Barker, TX). Target gene expression was measured using SYBR green PCR master mix (ThermoFisher, Waltham, MA) on an ABI 7500 Fast Real-Time PCR System. mRNA quantity was determined by standard curve analysis and the data are reported as a ratio of the quantity of the target gene divided by the quantity of a housekeeping gene. The human primer sets used were as follows: CYP24A1 forward 5′-CCGTAGCCTTCTTTGCGG-3′ and reverse 5′-CCCAGCGGCTGGAGATC-3′, TRPV6 forward 5′-CAGGGCCTGGACATCATTA-3′ and reverse 5′-AGA GCCGAGATGAGCAGAAC-3′, TMPRSS2 forward 5′-AGGATCGGTGTGTTCGCCTC-3′ and reverse 5′-CTCGTTCCAGTCGTCTTGGC-3′, ERG forward 5′-CACCGAACGAGCGCAGAGTT-3′ and reverse 5′-ACTGCCGCACATGGTCTGTA-3′. The mouse primer sets were as follows: Cyp24a1 forward 5′-AAGAACTGTA CGCTGCTGTCAC-3′ and reverse 5′-GGGATTCCGGGA TAGATTGTAG-3′, β-2-microglobulin (β2m) forward 5′-TCCAGAAAACCCCTCAAATTCAAG-3′ and reverse 5′-CAGTATGTTCGGCTTCCCATTC-3′. Primer sets were purchased from Sigma-Aldrich (St. Louis, MO).

### Animal studies

20 male NCr nude *sp/sp* mice 5 weeks of age were purchased from Taconic (Cambridge City, Indiana). The mice were housed and treated in accordance with IACUC regulations and the NIH Care and Use Guide in the transgenic mouse facility at Baylor College of Medicine. Mice were transitioned to an AIN-93 diet modified to contain 200 IU/kg of vitamin D that gives serum 25-hydroxyvitamin D levels similar to human levels [12] from Research Diets Inc. (New Brunswick, NJ).

### Xenograft tumors

VCaP cells were trypsinized with 0.25% Trypsin-EDTA (ThermoFisher, Waltham, MA) and mixed with HPS-19I prostate stromal cells [70] at a ratio of 4:1 with 2 million VCaP cells, 500,000 HPS-19I cells, and phenol red free Matrigel (Corning, Tewksbury, MA) using the DRS model previously described [29]. The cells were injected subcutaneously into the left and right flank of the mice.

### Treatments

Mice were treated 5 days per week via a 22-gauge gavage needle with 50 μl of vehicle (EtOH) or VDRM2 3 μg/kg of body weight dissolved in sesame oil.

### Measurements

Mice were weighed twice per week. Once the tumors were palpable, they were measured using digital calipers once per week. Tumor volume was calculated using a modified ellipsoid volume formula: *pi/6 x (L x W)^3/2^*. One-Way ANOVA with Tukey's multiple comparisons test was used to analyze differences in mouse weight. Two-Way ANOVA with Sidak's multiple comparisons test was used to analyze differences in tumor volume.

### Serum calcium analysis

Animals were anesthetized using Isoflurane, USP (Abbott Labs, Irving, Texas) and blood was collected via cardiac puncture at the completion of the study. Blood was allowed to clot at room temperature for at least 30 minutes. The blood was centrifuged at 13,000 rpm for 1 min to separate the serum. Serum was collected using a glass Pasteur pipette and transferred to a fresh Eppendorf tube. Serum samples were snap frozen in liquid nitrogen and stored at −80°C. Serum was submitted to the Baylor College of Medicine Pathology Core for serum calcium analysis. An unpaired, two-tailed *t-test* was used to analyze differences in serum calcium levels.

### Tumor collection

At the completion of the study, mice were anesthetized using Isoflurane, USP. Once the animals were deeply anesthetized, cardiac exsanguination was performed to collect blood followed by cervical dislocation as a secondary method to ensure death. The tumors were measured a final time with digital calipers, and the tumor mass was determined after resection. An unpaired, two-tailed *t-test* was used to analyze differences in tumor mass.

### Kidney RNA extraction

Kidneys were collected, snap frozen in liquid nitrogen, and stored at -80°C. Kidneys were crushed on dry ice with a metal mallet and transferred to individual 2 ml tubes containing Lysing Matrix D (MP Biomedicals, Santa Ana, California). One ml of TriReagent was added and kidneys were homogenized using a TissueLyser LT (Qiagen, Germantown, MD). In order to ensure complete homogenization, lysates were added to QIAShredder columns (Qiagen, Germantown, MD) and centrifuged at 12,000 × g for 1 minute. The aqueous phase was extracted by adding 200 μl of chloroform to the lysate followed by shaking and centrifugation at 12,000 × g for 15 minutes at 4°C. Total RNA was purified using the RNeasy RNA Isolation Kit (Qiagen, Germantown, MD). An unpaired, two-tailed *t-test* was used to analyze differences in kidney RNA levels.

### RNA sequencing

VCaP cells were plated at 400,000 cells per well in 6-well plates and allowed to adhere for 72 hours. Medium was replenished and the cells were treated with vehicle (EtOH), 10 nM 1,25D(OH)_2_D_3_, or 1 μM VDRM2 for 24 hours. Three biological replicates were used for the RNA sequencing. Total RNA was extracted using the RNeasy RNA Isolation Kit. RNA quality was assessed using an Agilent 2100 Bioanalyzer (Agilent Technologies, Santa Clara, CA) and all samples passed RIN and 28S:18S ratio analysis. Library construction consisted of a 200 bp short-insert library and read length was 100 paired-end sequencing with 50 million reads per sample. Detection of sequencing fragments was via the Illumina HiSeq2000 (Illumina, San Diego, CA). Sequencing data was mapped using TopHat2 [71] onto the human genome, genome build UCC hg19, and gene expression was assessed using Cufflinks2 [72]. Significantly changed genes were determined using the R statistical system; significance was assessed using ANOVA, and the Benjamini-Hochberg method was employed for multiple hypothesis testing correction. Significance was assessed at *q*-value < 0.25. The *q*-value is a false discovery rate (FDR) adjusted *p-value*. Enriched pathways were determined using the Gene Set Enrichment (GSEA) method [73], and the gene set collection from the Molecular Signature Database (MSigDB). Heat maps were generated using the R statistical system.

### Statistical analysis

All cell experiments contained three independent samples per treatment and each experiment was completed a minimum of three times unless otherwise indicated. All cell data are plotted as mean ± SEM (standard error of the mean) and are representative of three independent experiments except Figure 1C–1D where two independent samples per condition were completed a minimum of three times and are averaged on the graph. One-way ANOVA with Tukey's or Sidak's multiple comparisons test were used to analyze cell experiment results with GraphPad Prism version 7 (GraphPad, La Jolla, CA) and considered to be statistically significant at *p <* 0.05.

## SUPPLEMENTARY MATERIALS FIGURES AND TABLES



## References

[1] Donkena KV, Young CY (2011). Vitamin d, sunlight and prostate cancer risk. Adv Prev Med.

[2] Loke TW, Seyfi D, Khadra M (2011). Prostate cancer incidence in Australia correlates inversely with solar radiation. BJU Int.

[3] Ahonen MH, Tenkanen L, Teppo L, Hakama M, Tuohimaa P (2000). Prostate cancer risk and prediagnostic serum 25-hydroxyvitamin D levels (Finland). Cancer Causes Control.

[4] Shui IM, Mucci LA, Kraft P, Tamimi RM, Lindstrom S, Penney KL, Nimptsch K, Hollis BW, Dupre N, Platz EA, Stampfer MJ, Giovannucci E (2012). Vitamin D-related genetic variation, plasma vitamin D, and risk of lethal prostate cancer: a prospective nested case-control study. J Natl Cancer Inst.

[5] Anic GM, Albanes D, Rohrmann S, Kanarek N, Nelson WG, Bradwin G, Rifai N, McGlynn KA, Platz EA, Mondul AM (2016). Association between serum 25-hydroxyvitamin D and serum sex steroid hormones among men in NHANES. Clin Endocrinol (Oxf).

[6] Jacobs ET, Kohler LN, Kunihiro AG, Jurutka PW (2016). Vitamin D, Colorectal, Breast, and Prostate Cancers: A Review of the Epidemiological Evidence. J Cancer.

[7] Mondul AM, Weinstein SJ, Moy KA, Mannisto S, Albanes D (2016). Circulating 25-Hydroxyvitamin D, Prostate Cancer Survival. Cancer Epidemiol Biomarkers Prev.

[8] Ahn J, Peters U, Albanes D, Purdue MP, Abnet CC, Chatterjee N, Horst RL, Hollis BW, Huang WY, Shikany JM, Hayes RB, Prostate LC, Ovarian Cancer Screening Trial Project T (2008). Serum vitamin D concentration and prostate cancer risk: a nested case-control study. J Natl Cancer Inst.

[9] Skowronski RJ, Peehl DM, Feldman D (1993). Vitamin D and prostate cancer: 1,25 dihydroxyvitamin D3 receptors and actions in human prostate cancer cell lines. Endocrinology.

[10] Washington MN, Weigel NL (2010). 1{alpha},25-Dihydroxyvitamin D3 inhibits growth of VCaP prostate cancer cells despite inducing the growth-promoting TMPRSS2: ERG gene fusion. Endocrinology.

[11] Washington MN, Kim JS, Weigel NL (2011). 1alpha,25-dihydroxyvitamin D3 inhibits C4-2 prostate cancer cell growth via a retinoblastoma protein (Rb)-independent G1 arrest. Prostate.

[12] Kovalenko PL, Zhang Z, Yu JG, Li Y, Clinton SK, Fleet JC (2011). Dietary vitamin D and vitamin D receptor level modulate epithelial cell proliferation and apoptosis in the prostate. Cancer Prev Res (Phila).

[13] Blutt SE, Polek TC, Stewart LV, Kattan MW, Weigel NL (2000). A calcitriol analogue, EB1089, inhibits the growth of LNCaP tumors in nude mice. Cancer Res.

[14] Lokeshwar BL, Schwartz GG, Selzer MG, Burnstein KL, Zhuang SH, Block NL, Binderup L (1999). Inhibition of prostate cancer metastasis in vivo: a comparison of 1,23-dihydroxyvitamin D (calcitriol) and EB1089. Cancer Epidemiol Biomarkers Prev.

[15] Polek TC, Murthy S, Blutt SE, Boehm MF, Zou A, Weigel NL, Allegretto EA (2001). Novel nonsecosteroidal vitamin D receptor modulator inhibits the growth of LNCaP xenograft tumors in athymic mice without increased serum calcium. Prostate.

[16] Srinivas S, Feldman D (2009). A phase II trial of calcitriol and naproxen in recurrent prostate cancer. Anticancer Res.

[17] Chadha MK, Tian L, Mashtare T, Payne V, Silliman C, Levine E, Wong M, Johnson C, Trump DL (2010). Phase 2 trial of weekly intravenous 1,25 dihydroxy cholecalciferol (calcitriol) in combination with dexamethasone for castration-resistant prostate cancer. Cancer.

[18] Beer TM, Myrthue A (2004). Calcitriol in cancer treatment: from the lab to the clinic. Mol Cancer Ther.

[19] Johnson CS, Muindi JR, Hershberger PA, Trump DL (2006). The antitumor efficacy of calcitriol: preclinical studies. Anticancer Res.

[20] Trump DL, Hershberger PA, Bernardi RJ, Ahmed S, Muindi J, Fakih M, Yu WD, Johnson CS (2004). Anti-tumor activity of calcitriol: pre-clinical and clinical studies. J Steroid Biochem Mol Biol.

[21] Tomlins SA, Rhodes DR, Perner S, Dhanasekaran SM, Mehra R, Sun XW, Varambally S, Cao X, Tchinda J, Kuefer R, Lee C, Montie JE, Shah RB (2005). Recurrent fusion of TMPRSS2 and ETS transcription factor genes in prostate cancer. Science.

[22] Wang J, Cai Y, Ren C, Ittmann M (2006). Expression of variant TMPRSS2/ERG fusion messenger RNAs is associated with aggressive prostate cancer. Cancer Res.

[23] Wang J, Cai Y, Yu W, Ren C, Spencer DM, Ittmann M (2008). Pleiotropic biological activities of alternatively spliced TMPRSS2/ERG fusion gene transcripts. Cancer Res.

[24] Tomlins SA, Laxman B, Varambally S, Cao X, Yu J, Helgeson BE, Cao Q, Prensner JR, Rubin MA, Shah RB, Mehra R, Chinnaiyan AM (2008). Role of the TMPRSS2-ERG gene fusion in prostate cancer. Neoplasia.

[25] Sun C, Dobi A, Mohamed A, Li H, Thangapazham RL, Furusato B, Shaheduzzaman S, Tan SH, Vaidyanathan G, Whitman E, Hawksworth DJ, Chen Y, Nau M (2008). TMPRSS2-ERG fusion, a common genomic alteration in prostate cancer activates C-MYC and abrogates prostate epithelial differentiation. Oncogene.

[26] Wu X, Gong S, Roy-Burman P, Lee P, Culig Z (2013). Current mouse and cell models in prostate cancer research. Endocr Relat Cancer.

[27] Kim JS, Roberts JM, Bingman WE, Shao L, Wang J, Ittmann MM, Weigel NL (2014). The prostate cancer TMPRSS2: ERG fusion synergizes with the vitamin D receptor (VDR) to induce CYP24A1 expression-limiting VDR signaling. Endocrinology.

[28] Sato M, Lu J, Iturria S, Stayrook KR, Burris LL, Zeng QQ, Schmidt A, Barr RJ, Montrose-Rafizadeh C, Bryant HU, Ma YL (2010). A nonsecosteroidal vitamin D receptor ligand with improved therapeutic window of bone efficacy over hypercalcemia. J Bone Miner Res.

[29] Tuxhorn JA, McAlhany SJ, Dang TD, Ayala GE, Rowley DR (2002). Stromal cells promote angiogenesis and growth of human prostate tumors in a differential reactive stroma (DRS) xenograft model. Cancer Res.

[30] Polek TC, Stewart LV, Ryu EJ, Cohen MB, Allegretto EA, Weigel NL (2003). p53 Is required for 1,25-dihydroxyvitamin D3-induced G0 arrest but is not required for G1 accumulation or apoptosis of LNCaP prostate cancer cells. Endocrinology.

[31] Rohan JN, Weigel NL (2009). 1Alpha,25-dihydroxyvitamin D3 reduces c-Myc expression, inhibiting proliferation and causing G1 accumulation in C4-2 prostate cancer cells. Endocrinology.

[32] Urbinati G, Ali HM, Rousseau Q, Chapuis H, Desmaele D, Couvreur P, Massaad-Massade L (2015). Antineoplastic Effects of siRNA against TMPRSS2-ERG Junction Oncogene in Prostate Cancer. PLoS One.

[33] DeLuca HF, Zierold C (1998). Mechanisms and functions of vitamin D. Nutr Rev.

[34] Harrell RM, Lyles KW, Harrelson JM, Friedman NE, Drezner MK (1985). Healing of bone disease in X-linked hypophosphatemic rickets/osteomalacia. Induction and maintenance with phosphorus and calcitriol. J Clin Invest.

[35] Eelen G, Gysemans C, Verlinden L, Vanoirbeek E, De Clercq P, Van Haver D, Mathieu C, Bouillon R, Verstuyf A (2007). Mechanism and potential of the growth-inhibitory actions of vitamin D and ana-logs. Curr Med Chem.

[36] Matsumoto T, Miki T, Hagino H, Sugimoto T, Okamoto S, Hirota T, Tanigawara Y, Hayashi Y, Fukunaga M, Shiraki M, Nakamura T (2005). A new active vitamin D, ED-71, increases bone mass in osteoporotic patients under vitamin D supplementation: a randomized, double-blind, placebo-controlled clinical trial. J Clin Endocrinol Metab.

[37] Leyssens C, Verlinden L, Verstuyf A (2014). The future of vitamin D analogs. Front Physiol.

[38] Swami S, Krishnan AV, Peehl DM, Feldman D (2005). Genistein potentiates the growth inhibitory effects of 1,25-dihydroxyvitamin D3 in DU145 human prostate cancer cells: role of the direct inhibition of CYP24 enzyme activity. Mol Cell Endocrinol.

[39] Tannour-Louet M, Lewis SK, Louet JF, Stewart J, Addai JB, Sahin A, Vangapandu HV, Lewis AL, Dittmar K, Pautler RG, Zhang L, Smith RG, Lamb DJ (2014). Increased expression of CYP24A1 correlates with advanced stages of prostate cancer and can cause resistance to vitamin D3-based therapies. FASEB J.

[40] Ly LH, Zhao XY, Holloway L, Feldman D (1999). Liarozole acts synergistically with 1alpha,25-dihydroxyvitamin D3 to inhibit growth of DU 145 human prostate cancer cells by blocking 24-hydroxylase activity. Endocrinology.

[41] Boehm MF, Fitzgerald P, Zou A, Elgort MG, Bischoff ED, Mere L, Mais DE, Bissonnette RP, Heyman RA, Nadzan AM, Reichman M, Allegretto EA (1999). Novel nonsecosteroidal vitamin D mimics exert VDR-modulating activities with less calcium mobilization than 1,25-dihydroxyvitamin D3. Chem Biol.

[42] Dusso AS, Negrea L, Gunawardhana S, Lopez-Hilker S, Finch J, Mori T, Nishii Y, Slatopolsky E, Brown AJ (1991). On the mechanisms for the selective action of vitamin D analogs. Endocrinology.

[43] Wolchok JD, Kluger H, Callahan MK, Postow MA, Rizvi NA, Lesokhin AM, Segal NH, Ariyan CE, Gordon RA, Reed K, Burke MM, Caldwell A, Kronenberg SA (2013). Nivolumab plus ipilimumab in advanced melanoma. N Engl J Med.

[44] Hamid O, Robert C, Daud A, Hodi FS, Hwu WJ, Kefford R, Wolchok JD, Hersey P, Joseph RW, Weber JS, Dronca R, Gangadhar TC, Patnaik A (2013). Safety and tumor responses with lambrolizumab (anti-PD-1) in melanoma. N Engl J Med.

[45] Coffelt SB, de Visser KE (2015). Immune-mediated mechanisms influencing the efficacy of anticancer therapies. Trends Immunol.

[46] Gandini S, Massi D, Mandala M (2016). PD-L1 expression in cancer patients receiving anti PD-1/PD-L1 antibodies: A systematic review and meta-analysis. Crit Rev Oncol Hematol.

[47] Kongsbak M, Levring TB, Geisler C, von Essen MR (2013). The vitamin d receptor and T cell function. Front Immunol.

[48] Sochorova K, Budinsky V, Rozkova D, Tobiasova Z, Dusilova-Sulkova S, Spisek R, Bartunkova J (2009). Paricalcitol (19-nor-1,25-dihydroxyvitamin D2) and calcitriol (1,25-dihydroxyvitamin D3) exert potent immunomodulatory effects on dendritic cells and inhibit induction of antigen-specific T cells. Clin Immunol.

[49] Barragan M, Good M, Kolls JK (2015). Regulation of Dendritic Cell Function by. Vitamin D. Nutrients.

[50] Sherman MH, Yu RT, Engle DD, Ding N, Atkins AR, Tiriac H, Collisson EA, Connor F, Van Dyke T, Kozlov S, Martin P, Tseng TW, Dawson DW (2014). Vitamin D receptor-mediated stromal reprogramming suppresses pancreatitis and enhances pancreatic cancer therapy. Cell.

[51] Billerbeck E, Barry WT, Mu K, Dorner M, Rice CM, Ploss A (2011). Development of human CD4+FoxP3+ regulatory T cells in human stem cell factor-, granulocyte-macrophage colony-stimulating factor-, and interleukin-3-expressing NOD-SCID IL2Rgamma(null) humanized mice. Blood.

[52] Tanaka S, Saito Y, Kunisawa J, Kurashima Y, Wake T, Suzuki N, Shultz LD, Kiyono H, Ishikawa F (2012). Development of mature and functional human myeloid subsets in hematopoietic stem cell-engrafted NOD/SCID/IL2rgammaKO mice. J Immunol.

[53] Ishikawa F, Yasukawa M, Lyons B, Yoshida S, Miyamoto T, Yoshimoto G, Watanabe T, Akashi K, Shultz LD, Harada M (2005). Development of functional human blood and immune systems in NOD/SCID/IL2 receptor {gamma} chain(null) mice. Blood.

[54] Wang WL, Chatterjee N, Chittur SV, Welsh J, Tenniswood MP (2011). Effects of 1alpha,25 dihydroxyvitamin D3 and testosterone on miRNA and mRNA expression in LNCaP cells. Mol Cancer.

[55] Guzey M, Luo J, Getzenberg RH (2004). Vitamin D3 modulated gene expression patterns in human primary normal and cancer prostate cells. J Cell Biochem.

[56] Khanim FL, Gommersall LM, Wood VH, Smith KL, Montalvo L, O’Neill LP, Xu Y, Peehl DM, Stewart PM, Turner BM, Campbell MJ (2004). Altered SMRT levels disrupt vitamin D3 receptor signalling in prostate cancer cells. Oncogene.

[57] Qiao S, Pennanen P, Nazarova N, Lou YR, Tuohimaa P (2003). Inhibition of fatty acid synthase expression by 1alpha,25-dihydroxyvitamin D3 in prostate cancer cells. J Steroid Biochem Mol Biol.

[58] Krishnan AV, Shinghal R, Raghavachari N, Brooks JD, Peehl DM, Feldman D (2004). Analysis of vitamin D-regulated gene expression in LNCaP human prostate cancer cells using cDNA microarrays. Prostate.

[59] Ikezoe T, Gery S, Yin D, O’Kelly J, Binderup L, Lemp N, Taguchi H, Koeffler HP (2005). CCAAT/enhancer-binding protein delta: a molecular target of 1,25-dihydroxyvitamin D3 in androgen-responsive prostate cancer LNCaP cells. Cancer Res.

[60] Boyle BJ, Zhao XY, Cohen P, Feldman D (2001). Insulin-like growth factor binding protein-3 mediates 1 alpha,25-dihydroxyvitamin d(3) growth inhibition in the LNCaP prostate cancer cell line through p21/WAF1. J Urol.

[61] Berger MF, Lawrence MS, Demichelis F, Drier Y, Cibulskis K, Sivachenko AY, Sboner A, Esgueva R, Pflueger D, Sougnez C, Onofrio R, Carter SL, Park K (2011). The genomic complexity of primary human prostate cancer. Nature.

[62] Barry M, Perner S, Demichelis F, Rubin MA (2007). TMPRSS2-ERG fusion heterogeneity in multifocal prostate cancer: clinical and biologic implications. Urology.

[63] Minner S, Gartner M, Freudenthaler F, Bauer M, Kluth M, Salomon G, Heinzer H, Graefen M, Bokemeyer C, Simon R, Sauter G, Schlomm T, Wilczak W (2013). Marked heterogeneity of ERG expression in large primary prostate cancers. Mod Pathol.

[64] Ayala G, Frolov A, Chatterjee D, He D, Hilsenbeck S, Ittmann M (2015). Expression of ERG protein in prostate cancer: variability and biological correlates. Endocr Relat Cancer.

[65] Tsourlakis MC, Stender A, Quaas A, Kluth M, Wittmer C, Haese A, Graefen M, Steurer S, Simon R, Korbel J, Weischenfeldt J, Huland H, Sauter G (2016). Heterogeneity of ERG expression in prostate cancer: a large section mapping study of entire prostatectomy specimens from 125 patients. BMC Cancer.

[66] Rowley DR, Tindall DJ (1987). Responses of NBT-II bladder carcinoma cells to conditioned medium from normal fetal urogenital sinus. Cancer Res.

[67] Jacobs ET, Van Pelt C, Forster RE, Zaidi W, Hibler EA, Galligan MA, Haussler MR, Jurutka PW (2013). CYP24A1 and CYP27B1 polymorphisms modulate vitamin D metabolism in colon cancer cells. Cancer Res.

[68] Agoulnik IU, Krause WC, Bingman WE, Rahman HT, Amrikachi M, Ayala GE, Weigel NL (2003). Repressors of androgen and progesterone receptor action. J Biol Chem.

[69] Bai W, Weigel NL (1996). Phosphorylation of Ser211 in the chicken progesterone receptor modulates its transcriptional activity. J Biol Chem.

[70] Kim W, Barron DA, San Martin R, Chan KS, Tran LL, Yang F, Ressler SJ, Rowley DR (2014). RUNX1 is essential for mesenchymal stem cell proliferation and myofibroblast differentiation. Proc Natl Acad Sci USA.

[71] Kim D, Pertea G, Trapnell C, Pimentel H, Kelley R, Salzberg SL (2013). TopHat2: accurate alignment of transcriptomes in the presence of insertions, deletions and gene fusions. Genome Biol.

[72] Trapnell C, Williams BA, Pertea G, Mortazavi A, Kwan G, van Baren MJ, Salzberg SL, Wold BJ, Pachter L (2010). Transcript assembly and quantification by RNA-Seq reveals unannotated transcripts and isoform switching during cell differentiation. Nat Biotechnol.

[73] Subramanian A, Tamayo P, Mootha VK, Mukherjee S, Ebert BL, Gillette MA, Paulovich A, Pomeroy SL, Golub TR, Lander ES, Mesirov JP (2005). Gene set enrichment analysis: a knowledge-based approach for interpreting genome-wide expression profiles. Proc Natl Acad Sci USA.

