# Supplementary material for: Vitamin D receptor activation reduces VCaP xenograft tumor growth and counteracts ERG activity despite induction of TMPRSS2:ERG
Source: Oncotarget. 2017 May 18;8(27):44447–64. doi: 10.18632/oncotarget.17968 (PMC5546493; doi:10.18632/oncotarget.17968)
Supplement: Supplementary file 1 [file oncotarget-08-44447-s001.pdf]

Vitamin D receptor activation reduces VCaP xenograft tumor growth and counteracts ERG activity despite induction of TMPRSS2:ERG

Supplementary Materials

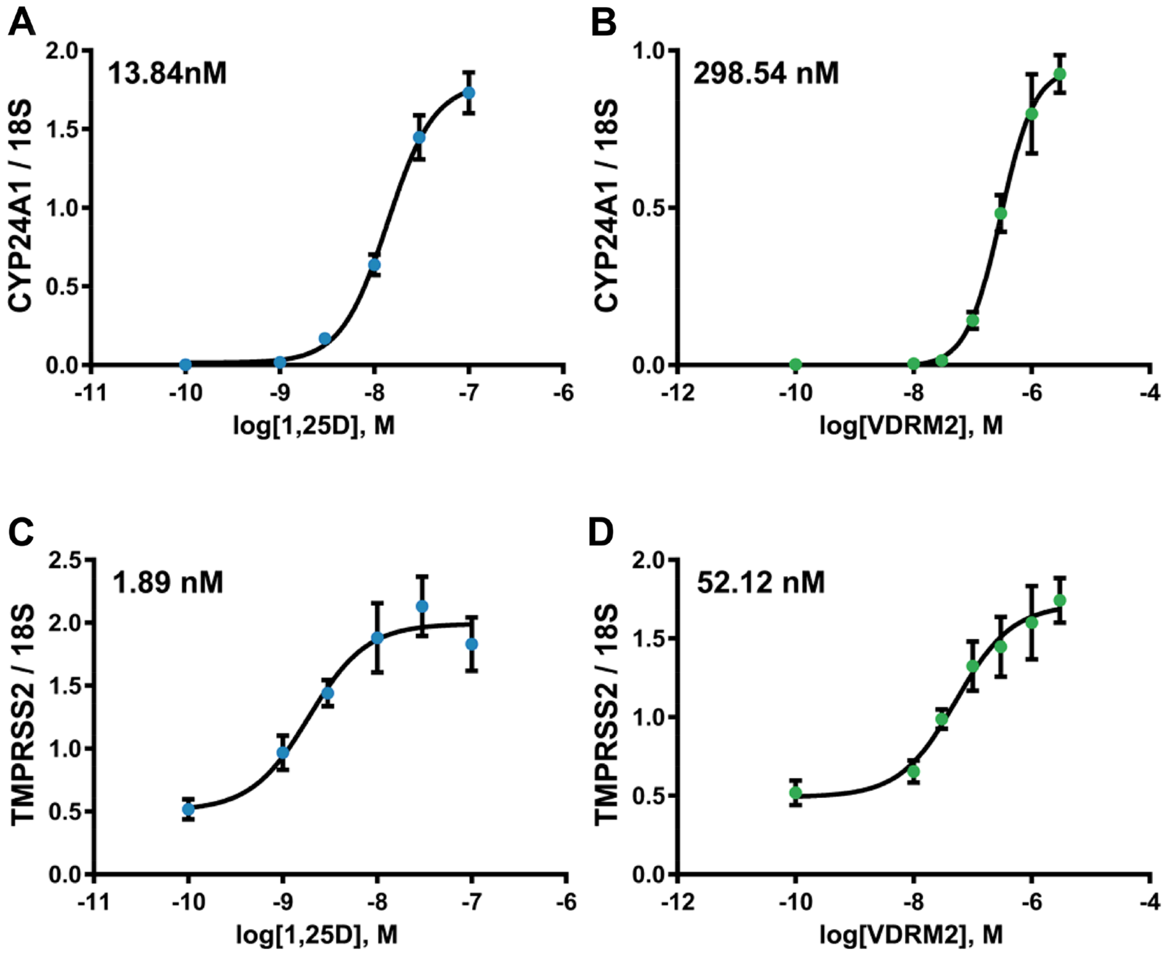

Supplementary Figure 1: Calculated EC_{50} values for VDR target genes CYP24A1 and TMPRSS2. (**A**) The EC_{50} value for 1,25D$(OH)_2D_3$ (1,25D)-mediated induction of CYP24A1 is 13.84 nM. (**B**) The EC_{50} value for VDRM2-mediated induction of CYP24A1 is 298.54 nM. (**C**) The EC_{50} value for 1,25D$(OH)_2D_3$-mediated induction of TMPRSS2 is 1.89 nM. (**D**) The EC_{50} value for VDRM2-mediated induction of TMPRSS2 is 52.12 nM.

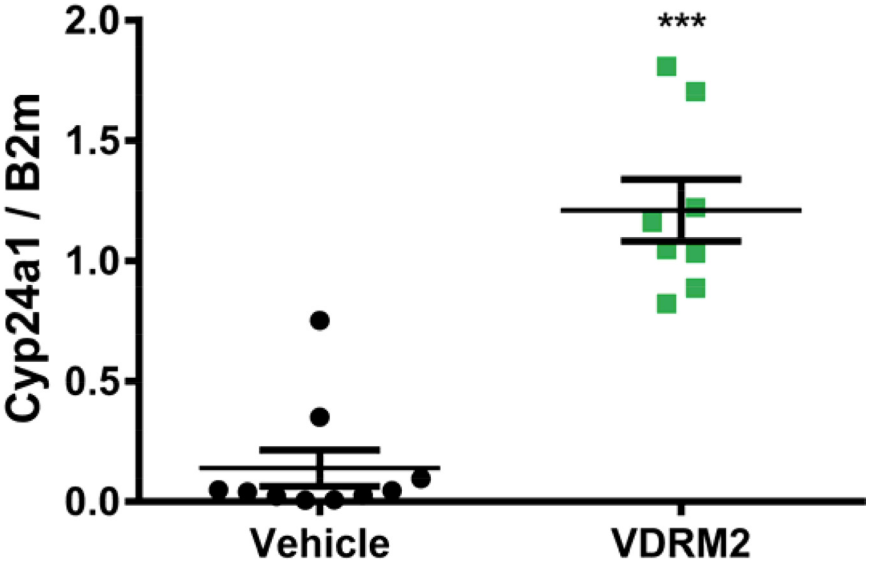

Supplementary Figure 2: VDRM2 induces Cyp24a1 in mouse kidneys. Kidneys were harvested from the mice and RNA was purified. VDR target gene Cyp24a1 mRNA was measured by q-PCR and normalized to β-2m. $n = 10$ vehicle, $n = 8$ VDRM2. ***$p < 0.001$, relative to vehicle control, mean ± SEM.

Supplementary Table 1: The "Unique" 1,25D(OH)$_2$D$_3$ upregulated genes from RNA sequencing

VCaP "Unique" 1,25D – Upregulated Genes (FC ≥ 1.5, q < 0.25)				
**Gene Symbol**	**Linear Fold Change 1,25D/Vehicle**	***p*-value 1,25D/Vehicle**	**Linear Fold Change VDRM2/Vehicle**	***p*-value VDRM2/Vehicle**
PYROXD2	***2.75***	***4.200E-03***	***0.73***	***2.139E-01***
SERPINA11	1.99	6.900E-03	1.36	1.316E-01
C1orf85	1.77	8.800E-03	1.32	1.710E-02
CRNKL1	1.60	8.300E-03	1.39	1.300E-02
SH3BGRL3	1.58	8.600E-03	1.48	1.300E-03
AP4S1	1.51	7.000E-04	1.40	1.490E-02
ACVR1	1.79	1.500E-03	1.40	4.750E-02
TESC	1.61	6.200E-03	1.47	5.600E-03
SMOX	1.50	5.800E-03	1.47	1.400E-02
SLC9A1	1.71	2.900E-03	1.50	2.400E-03
HLA-DMB	1.60	7.700E-03	1.36	1.250E-02
DDX58	1.61	5.000E-04	1.35	7.370E-02
RP11-343C2.8	1.96	1.410E-02	1.11	6.130E-01
INPP1	1.85	4.900E-03	1.45	1.200E-02
KLK15	1.83	4.400E-03	1.49	3.680E-02
MBOAT2	1.52	8.500E-03	1.36	1.410E-02
MCCC2	1.54	4.400E-03	1.35	3.680E-02
THSD7A	2.09	3.000E-04	1.26	2.255E-01
RFX6	1.53	5.000E-04	1.43	3.510E-02
NSMAF	1.67	4.100E-03	1.32	8.400E-02

VCaP cells were treated for 24 hours with vehicle (EtOH), 10 nM 1,25D(OH)$_2$D$_3$ (1,25D), or 1 µM VDRM2 then RNA was purified. Transcriptomic profiling was assessed using RNA-Seq on an Illumina HiSeq2000. Significance was assessed using ANOVA, followed by the Benjamini-Hochberg method for multiple hypothesis testing correction. Significance was assessed at *q*-value < 0.25. The genes that are "uniquely" upregulated by 1,25D(OH)$_2$D$_3$ compared to expression level in VDRM2-treated cells. Genes regulated in the opposite direction are bolded.

Supplementary Table 2: The "Unique" VDRM2 upregulated genes from RNA sequencing

VCaP "Unique" VDRM2 – Upregulated Genes (FC ≥ 1.5, q < 0.25)				
**Gene Symbol**	**Linear Fold Change 1,25D/Vehicle**	***p*-value 1,25D/Vehicle**	**Linear Fold Change VDRM2/Vehicle**	***p*-value VDRM2/Vehicle**
FZD1	1.47	8.800E-03	1.59	8.200E-03
JUNB	1.46	1.240E-02	1.56	3.600E-03
RND3	***0.69***	***6.863E-01***	***5.00***	***2.210E-02***
LACE1	1.09	3.742E-01	1.58	6.100E-03
TCP11L1	1.36	3.390E-02	1.63	5.500E-03
BAMBI	1.07	3.300E-03	1.52	1.420E-02
RP11-411B6.6	1.17	8.732E-01	5.56	1.210E-02
ZBTB16	1.33	2.460E-02	1.57	1.300E-02
TMEM63B	1.44	6.700E-03	1.64	2.500E-03
ZNF673	1.45	6.090E-02	1.92	3.600E-03
CA12	1.45	2.600E-03	1.60	1.100E-03
ZNF765	1.45	1.410E-02	1.51	7.700E-03
OSGIN1	1.38	1.400E-02	1.54	1.680E-02
PREX1	1.37	1.710E-02	1.62	1.020E-02
SLC25A30	1.27	8.690E-02	1.77	1.000E-04
LMLN	1.25	1.199E-01	1.60	2.500E-03
TNFAIP1	1.25	8.800E-03	1.53	6.000E-04
SWAP70	1.24	1.136E-01	1.56	3.900E-03

VCaP cells were treated for 24 hours with vehicle (EtOH), 10 nM 1,25D(OH)$_2$D$_3$ (1,25D), or 1 μM VDRM2 then RNA was purified. Transcriptomic profiling was assessed using RNA-Seq on an Illumina HiSeq2000. Significance was assessed using ANOVA, followed by the Benjamini-Hochberg method for multiple hypothesis testing correction. Significance was assessed at *q*-value < 0.25. The genes that are "uniquely" upregulated by VDRM2 compared to expression level in 1,25D(OH)$_2$D$_3$-treated cells. Genes regulated in the opposite direction are bolded.

Supplementary Table 3: The "Unique" $1,25D(OH)_2D_3$ downregulated genes from RNA sequencing

VCaP "Unique" 1,25D – Downregulated Genes (FC ≤ 0.667, q < 0.25)				
**Gene Symbol**	**Linear Fold Change 1,25D/Vehicle**	***p*-value 1,25D/Vehicle**	**Linear Fold Change VDRM2/Vehicle**	***p*-value VDRM2/Vehicle**
ADAMTS3	0.60	1.110E-02	0.69	1.980E-02
PANK1	0.62	1.300E-03	0.69	3.000E-04
DUS2L	0.56	1.560E-02	0.73	6.400E-03
ZNF239	0.64	8.000E-04	0.68	1.100E-02
ARHGEF4	0.63	7.700E-03	0.84	1.557E-01
TRERF1	0.65	1.000E-02	0.70	1.960E-02
RASL10B	0.66	9.000E-04	0.76	1.140E-02
NABP1	0.55	3.100E-03	0.84	4.060E-02
PAX1	0.62	1.500E-03	0.67	2.680E-02
C19orf44	0.66	1.470E-02	0.84	8.120E-02
AMT	***0.39***	***2.750E-02***	***1.36***	***1.182E-01***

VCaP cells were treated for 24 hours with vehicle (EtOH), 10 nM $1,25D(OH)_2D_3$ (1,25D), or 1 μM VDRM2 then RNA was purified. Transcriptomic profiling was assessed using RNA-Seq on an Illumina HiSeq2000. Significance was assessed using ANOVA, followed by the Benjamini-Hochberg method for multiple hypothesis testing correction. Significance was assessed at *q*-value < 0.25. The genes that are "uniquely" downregulated by $1,25D(OH)_2D_3$ compared to expression level in VDRM2-treated cells. Genes regulated in the opposite direction are bolded.

Supplementary Table 4: The "Unique" VDRM2 downregulated genes from RNA sequencing

VCaP "Unique" VDRM2 – Downregulated Genes (FC ≤ 0.667, q < 0.25)				
**Gene Symbol**	**Linear Fold Change 1,25D/Vehicle**	***p*-value 1,25D/Vehicle**	**Linear Fold Change VDRM2/Vehicle**	***p*-value VDRM2/Vehicle**
DUS3L	0.68	9.300E-03	0.67	8.100E-03
NLE1	0.72	1.240E-02	0.66	3.000E-03
CRYM	0.67	2.700E-03	0.52	2.300E-03
SEZ6	0.77	1.300E-02	0.61	1.550E-02
PHGDH	0.72	8.100E-03	0.65	5.700E-03
C1R	***1.22***	***2.590E-02***	***0.59***	***2.500E-03***
ENHO	0.76	2.540E-02	0.41	8.600E-03
TMEM62	0.81	5.400E-03	0.64	8.500E-03
FAM134B	0.70	1.300E-03	0.59	9.000E-04
POLR2M	0.70	8.600E-03	0.62	1.670E-02
MEX3B	0.67	8.800E-03	0.62	5.000E-03
IMP3	0.76	4.500E-02	0.67	5.500E-03
ATP8B2	0.71	9.600E-03	0.64	9.000E-03
PDK3	0.69	1.440E-02	0.65	5.700E-03
GARS	0.72	6.800E-03	0.65	1.260E-02
HMBS	0.69	1.000E-04	0.66	1.970E-02
LPCAT4	0.79	8.000E-04	0.59	1.000E-04
C17orf90	0.80	2.730E-02	0.61	1.480E-02
C1orf233	0.69	6.900E-03	0.59	1.300E-03
POLR3C	0.70	2.100E-03	0.64	0.000E+00
C3orf78	0.91	3.883E-01	0.39	1.580E-02

VCaP cells were treated for 24 hours with vehicle (EtOH), 10 nM 1,25D$(OH)_2D_3$ (1,25D), or 1 µM VDRM2 then RNA was purified. Transcriptomic profiling was assessed using RNA-Seq on an Illumina HiSeq2000. Significance was assessed using ANOVA, followed by the Benjamini-Hochberg method for multiple hypothesis testing correction. Significance was assessed at *q*-value < 0.25. The genes that are "uniquely" downregulated by VDRM2 compared to expression level in 1,25D$(OH)_2D_3$-treated cells. Genes regulated in the opposite direction are bolded.

Supplementary Table 5: The top 30 genes upregulated by 1,25D(OH)$_2$D$_3$

VCaP Top 30 1,25D Upregulated Genes (FC ≥ 1.5, q < 0.25)		
**Gene Symbol**	**Linear Fold Change 1,25D/Vehicle**	***p*-value 1,25D/Vehicle**
CYP24A1	3218.89	3.024E-04
SULT1C2	53.62	5.513E-03
C8orf4	20.85	4.818E-05
ZNF516	19.57	1.821E-03
SPARC	19.22	8.640E-04
F3	14.12	6.320E-05
SNTB1	12.89	1.452E-03
LY96	10.24	4.033E-02
THBD	10.23	1.090E-03
MYO1G	9.21	4.741E-03
NFATC2	8.98	6.062E-04
TMPRSS2	8.94	1.709E-03
HSD3B1	8.31	6.866E-03
SCUBE2	7.76	6.874E-04
GDF15	7.74	1.328E-03
AMACR	7.01	5.880E-03
GOLGA7B	5.96	1.103E-04
SYTL5	5.48	8.617E-03
CEBPD	5.35	1.757E-03
ZNF703	5.34	3.685E-04
DCLK1	5.00	3.231E-03
C1orf87	4.99	3.903E-04
TLR3	4.85	3.652E-05
ARHGAP31	4.45	9.688E-03
SLC44A5	4.39	9.947E-05
TRPV6	4.29	1.697E-03
PTRF	4.25	1.530E-04
KANK4	3.78	2.948E-03
CDH12	3.73	1.600E-03
DHRS3	3.61	9.320E-04

VCaP cells were treated for 24 hours with vehicle (EtOH) or 10 nM 1,25D(OH)$_2$D$_3$ (1,25D) then RNA was purified. Transcriptomic profiling was assessed using RNA-Seq on an Illumina HiSeq2000. Significance was assessed using ANOVA, followed by the Benjamini-Hochberg method for multiple hypothesis testing correction. Significance was assessed at *q*-value < 0.25. The top 30 1,25D(OH)$_2$D$_3$ upregulated genes sorted by fold change, cutoff 1.5-fold regulation.

Supplementary Table 6: The top 30 genes downregulated by 1,25D(OH)$_2$D$_3$

VCaP Top 30 1,25D Downregulated Genes (FC ≤ 0.667, q < 0.25)		
**Gene Symbol**	**Linear Fold Change 1,25D/Vehicle**	***p*-value 1,25D/Vehicle**
NGEF	0.24	1.077E-02
MEIS1	0.26	4.795E-03
OPRK1	0.33	2.186E-03
SERP2	0.33	7.632E-03
MUCL1	0.35	1.527E-02
RORB	0.35	1.046E-02
GLYATL1	0.38	2.312E-02
AMT	0.39	2.749E-02
MALL	0.42	1.290E-04
NR4A2	0.43	1.444E-03
CSRP2	0.44	7.706E-03
STON2	0.44	1.947E-03
FAM84A	0.46	1.834E-02
LRRC3	0.46	7.189E-03
HMGCS2	0.46	4.602E-03
DDN	0.47	1.120E-04
WNT2	0.49	2.747E-03
MIR3654	0.49	3.139E-03
PEG3	0.49	1.501E-02
SERPINI1	0.51	6.058E-03
TSHZ1	0.53	5.144E-03
KLF5	0.53	1.050E-02
PARP8	0.54	8.781E-03
PLCE1	0.54	4.109E-02
PEG10	0.55	1.600E-03
TLL2	0.55	8.179E-03
NABP1	0.55	3.135E-03
DUS2L	0.56	1.555E-02
ANXA6	0.56	2.082E-03
SOX13	0.57	1.207E-03

VCaP cells were treated for 24 hours with vehicle (EtOH) or 10 nM 1,25D(OH)$_2$D$_3$ (1,25D) then RNA was purified. Transcriptomic profiling was assessed using RNA-Seq on an Illumina HiSeq2000. Significance was assessed using ANOVA, followed by the Benjamini-Hochberg method for multiple hypothesis testing correction. Significance was assessed at *q*-value < 0.25. The top 30 1,25D(OH)$_2$D$_3$ downregulated genes sorted by fold change, cutoff 1.5-fold regulation.

Supplementary Table 7: The top 30 genes upregulated by VDRM2

VCaP Top 30 VDRM2 Upregulated Genes (FC ≥ 1.5, q < 0.25)		
**Gene Symbol**	**Linear Fold Change VDRM2 / Vehicle**	***p*-value VDRM2 / Vehicle**
CYP24A1	7509.24	6.927E-04
SULT1C2	61.99	1.317E-02
ZNF516	25.08	8.851E-05
C8orf4	21.26	7.365E-04
SPARC	18.69	1.063E-03
LY96	17.28	5.819E-03
F3	16.40	5.355E-05
SNTB1	15.16	2.563E-03
TMPRSS2	10.84	2.045E-03
NFATC2	10.08	4.791E-05
GDF15	8.94	3.225E-03
MYO1G	8.76	1.532E-03
THBD	8.24	1.086E-03
SCUBE2	7.98	2.712E-04
AMACR	7.45	2.447E-03
HSD3B1	7.37	2.366E-03
LOXL2	6.68	1.129E-03
GOLGA7B	6.67	2.729E-04
ZNF703	6.23	6.212E-03
SYTL5	5.87	9.369E-03
RP11-411B6.6	5.56	1.214E-02
TRPV6	5.55	4.881E-03
DCLK1	5.07	7.137E-04
CEBPD	5.05	6.948E-04
RND3	5.00	2.211E-02
PTRF	4.92	8.637E-04
FAM131B	4.61	3.052E-03
SLC44A5	4.58	6.540E-03
C1orf87	4.58	2.650E-02
TLR3	4.55	8.849E-05

VCaP cells were treated for 24 hours with vehicle (EtOH) or 1 μM VDRM2 then RNA was purified. Transcriptomic profiling was assessed using RNA-Seq on an Illumina HiSeq2000. Significance was assessed using ANOVA, followed by the Benjamini-Hochberg method for multiple hypothesis testing correction. Significance was assessed at *q*-value < 0.25. The top 30 VDRM2 upregulated genes sorted by fold change, cutoff 1.5-fold regulation.

Supplementary Table 8: The top 30 genes downregulated by VDRM2

VCaP Top 30 VDRM2 Downregulated Genes (FC ≤ 0.667, q < 0.25)		
**Gene Symbol**	**Linear Fold Change VDRM2/Vehicle**	***p*-value VDRM2/Vehicle**
OPRK1	0.27	2.788E-03
MALL	0.35	1.827E-04
NR4A2	0.35	9.844E-03
GLYATL1	0.36	2.138E-02
MUCL1	0.36	9.873E-03
PARP8	0.37	1.533E-05
C3orf78	0.39	1.582E-02
RORB	0.39	1.565E-02
ENHO	0.41	8.597E-03
CSRP2	0.41	7.821E-03
FAM84A	0.42	7.019E-03
PLCE1	0.45	2.052E-02
MEIS1	0.45	1.030E-02
NGEF	0.46	4.499E-02
TSHZ1	0.46	5.435E-04
ADM	0.47	7.204E-03
WNT2	0.47	1.813E-03
FABP5	0.49	4.780E-03
SERP2	0.49	3.070E-02
HMGCS2	0.51	2.288E-03
CRYM	0.52	2.252E-03
BEND3	0.52	4.717E-03
SERPINI1	0.52	7.647E-03
DDN	0.53	2.509E-03
TLE1	0.53	7.263E-03
PEG10	0.55	5.005E-04
LIN7A	0.55	1.142E-03
PEG3	0.56	3.360E-02
SOX13	0.57	1.919E-03
ANXA6	0.59	2.425E-04

VCaP cells were treated for 24 hours with vehicle (EtOH) or 1 μM VDRM2 RNA was purified. Transcriptomic profiling was assessed using RNA-Seq on an Illumina HiSeq2000. Significance was assessed using ANOVA, followed by the Benjamini-Hochberg method for multiple hypothesis testing correction. Significance was assessed at *q*-value < 0.25. The top 30 VDRM2 downregulated genes sorted by fold change, cutoff 1.5-fold regulation.

Supplementary Table 9: Normalized enrichment scores and *q*-values from Gene Set Enrichment Analysis (GSEA) of the top 5 Hallmark gene set collection pathways regulated by 1,25D(OH)$_2$D$_3$ and VDRM2

Gene Set Enrichment Analysis Top 5 Hallmark Gene Set Pathways				
	1,25D		VDRM2	
**HALLMARK Gene Set**	**NES**	**-Log10 (q-value)**	**NES**	**-Log10 (*q*-value)**
MYC TARGETS V1	−4.87	> 4.0	−5.12	> 4.0
E2F TARGETS	−4.63	> 4.0	−4.50	> 4.0
MYC TARGETS V2	−4.16	> 4.0	−3.96	> 4.0
G2M CHECKPOINT	−3.51	> 4.0	−3.11	> 4.0
ANDROGEN RESPONSE	3.03	4.10	3.07	3.14

NES: Normalized enrichment score.The top 5 pathways significantly regulated via GSEA using the Hallmark gene set collection comparing 1,25D(OH)$_2$D$_3$ (1,25D) and VDRM2-treated VCaP cells. NES: normalized enrichment score. *q*-value: False discovery rate (FDR) adjusted *p*-value.

Supplementary Table 10: Normalized enrichment scores and q-values from Gene Set Enrichment Analysis (GSEA) of the top 10 Hallmark gene set collection pathways regulated by ERG, 1,25D(OH)$_2$D$_3$, and VDRM2

Gene Set Enrichment Analysis Top 10 Hallmark Gene Set Pathways						
	siERG IVB		1,25D		VDRM2	
**HALLMARK Gene Set**	**NES**	**-Log10 (*q*-value)**	**NES**	**-Log10 (*q*-value)**	**NES**	**-Log10 (*q*-value)**
MYC TARGETS V1	3.07	2.82	−4.87	> 4.0	−5.12	> 4.0
MYC TARGETS V2	2.68	2.44	−4.16	> 4.0	−3.96	> 4.0
E2F TARGETS	2.55	2.18	−4.63	> 4.0	−4.50	> 4.0
G2M CHECKPOINT	2.35	1.69	−3.51	> 4.0	−3.11	> 4.0
INF GAMMA RESPONSE	−2.64	2.01	-	N.S.	-	N.S.
INF ALPHA RESPONSE	−2.48	1.91	-	N.S.	-	N.S.
INFLAMMATORY RESPONSE	−1.97	0.99	2.19	1.65	1.97	1.07
HEME METABOLISM	−3.09	2.90	1.67	0.81	1.62	0.73
PROTEIN SECRETION	−2.26	1.46	2.82	3.40	2.83	2.56
KRAS SIGNALING UP	−2.19	1.36	2.06	1.39	1.81	0.87

NES: Normalized enrichment score. The top 10 pathways significantly regulated via GSEA using the Hallmark gene set collection comparing siERG IVB, 1,25D(OH)$_2$D$_3$ (1,25D) and VDRM2-treated VCaP cells. NES: normalized enrichment score. *q*-value: False discovery rate (FDR) adjusted *p*-value.
